# MCL attenuates atherosclerosis by suppressing macrophage ferroptosis via targeting KEAP1/NRF2 interaction

**DOI:** 10.1016/j.redox.2023.102987

**Published:** 2023-12-07

**Authors:** Xing Luo, Yuehong Wang, Xinxin Zhu, Yuwu Chen, Biyi Xu, Xiaoxuan Bai, Xiuzhu Weng, Jinmei Xu, Yangyang Tao, Dan Yang, Jie Du, Ying Lv, Shan Zhang, Sining Hu, Ji Li, Haibo Jia

**Affiliations:** a Department of Cardiology, 2nd Affiliated Hospital of Harbin Medical University, Harbin, 150001, PR China; b National Key Laboratory of Frigid Zone Cardiovascular Diseases; The Key Laboratory of Myocardial Ischemia, Chinese Ministry of Education, Harbin 150001, PR China; c State Key Laboratory of Systems Medicine for Cancer, Division of Cardiology, Renji Hospital, School of Medicine, Shanghai Jiao Tong University, Shanghai, Cancer Institute, Shanghai, 200127, PR China; d Department of Endocrinology, Fourth Affiliated Hospital of Harbin Medical University, Harbin, 150001, PR China; e Department of Ultrasound, 2nd Affiliated Hospital of Harbin Medical University, Harbin, 150001, PR China; f Department of Forensic Medicine, Harbin Medical University, Harbin, 150001, PR China

**Keywords:** Atherosclerosis, MCL, Ferroptosis, KEAP1/NRF2, Oxidative stress

## Abstract

**Background:**

Micheliolide (MCL), which is the active metabolite of parthenolide, has demonstrated promising clinical application potential. However, the effects and underlying mechanisms of MCL on atherosclerosis are still unclear.

**Method:**

ApoE^−/−^ mice were fed with high fat diet, with or without MCL oral administration, then the plaque area, lipid deposition and collagen content were determined. *In vitro*, MCL was used to pretreat macrophages combined by ox-LDL, the levels of ferroptosis related proteins, NRF2 activation, mitochondrial function and oxidative stress were detected.

**Results:**

MCL administration significantly attenuated atherosclerotic plaque progress, which characteristics with decreased plaque area, less lipid deposition and increased collagen. Compared with HD group, the level of GPX4 and xCT in atherosclerotic root macrophages were increased in MCL group obviously. *In vitro* experiment demonstrated that MCL increased GPX4 and xCT level, improved mitochondrial function, attenuated oxidative stress and inhibited lipid peroxidation to suppress macrophage ferroptosis induced with ox-LDL. Moreover, MCL inhibited KEAP1/NRF2 complex formation and enhanced NRF2 nucleus translocation, while the protective effect of MCL on macrophage ferroptosis was abolished by NRF2 inhibition. Additionally, molecular docking suggests that MCL may bind to the Arg483 site of KEAP1, which also contributes to KEAP1/NRF2 binding. Furthermore, Transfection Arg483 (KEAP1-R483S) mutant plasmid can abrogate the *anti*-ferroptosis and anti-oxidative effects of MC in macrophages. KEAP1-R483S mutation also limited the protective effect of MCL on atherosclerosis progress and macrophage ferroptosis in ApoE^−/−^ mice.

**Conclusion:**

MCL suppressed atherosclerosis by inhibiting macrophage ferroptosis via activating NRF2 pathway, the related mechanism is through binding to the Arg483 site of KEAP1 competitively.

## Highlights

• MCL attenuated AS development, decreased inflammatory response and improved oxidative stress.
• MCL inhibited macrophage ferroptosis and mitochondrial function to attenuate the progress of AS.
• MCL suppressed ferroptosis by increasing GPX4 and xCT via activating NRF2 pathway.
• MCL promotes KEAP1/NRF2 dissociation by competing with NRF2 to bind the Arg483 site of KEAP1.

## Abbreviations

AAV: Adeno-associated virus
AS: Atherosclerosis
CCK8: Cell Counting Kit
GPX4: Glutathione peroxidase4
GSH: Glutathione
GBM: Glioblastoma
PFN: ferroportin
ROS: Reactive oxygen species
NRF2: Nuclear factor erythroid -related factor 2
KEAP1: Kelch-1ike ECH- associated protein l
AAV: Adeno-associated virus
NC: Negative control
TEM: Transmission electron microscopy
MDA: Malondialdehyde
HD: High fat diet
ND: Normal fat diet
KEAP1-WT: KEAP1 wild type
qPCR: quantitative polymerase chain reaction
xCT: Xap5 circadian timekeeper

## 1 Introduction

Atherosclerosis (AS) is a leading cause of the progress in cardiovascular disease worldwide, whose major clinical manifestations including ischemic heart disease, stroke, and peripheral arterial disease [1]. During the development of AS, substantial lipid deposition and necrotic core increase the instability of plaque, promote plaque rupture and thrombus formation. Nowadays, despite great progress have been made in aspect of lipid-lowering and anti-inflammatory treatments, a large percentage of patients suffering from AS is still at the risk of ischemic events. Thus, the discovery of new therapeutic targets and drugs for AS is urgently needed.

In the advanced stage of AS, it was observed a large amount of macrophage death in the plaques. Macrophage death leads to the increased inflammatory response and efferocytosis dysfunction, which enhanced the formation of atherosclerotic necrotic core [2,3]. Previous study reported that GSDME-mediated macrophage pyroptosis promotes the progression and associated inflammation of AS [4]. Furthermore, it was also reported that inhibiting macrophage death could improve inflammatory response and attenuate the development of AS [5,6]. This evidence suggested that macrophage death may be a potential target for prevent of AS progression.

Ferroptosis is a newly discovered type of programmed death, which is regulated by multiple cellular metabolic pathways, including imbalance of redox homeostasis, iron handling, mitochondrial dysfunction and amino acids metabolic disorder [7]. Intracellular oxidative stress imbalance and lipid peroxidation of cell membrane are the most important characteristics of ferroptosis [8,9]. Recently, in patients with atherosclerotic plaques, the genes responsible for ferroptosis were found to be significantly altered [10]. Furthermore, it was also reported that inhibition of ferroptosis alleviates AS via attenuating lipid peroxidation [11]. Additionally, our previous study also found that cigarette smoking promoting AS progress by enhancing macrophage ferroptosis [12].

Lipid peroxidation of cell membrane represents the crucial characteristic of ferroptosis. In detail, anti-oxidant system represented by Xap5 circadian timekeeper (xCT), glutathione (GSH) and glutathione peroxidase4 (GPX4) is unable to remove excessive intracellular lipid reactive oxygen species (ROS) [13]. The main function of xCT is to induce cystine for glutathione biosynthesis, while GPX4 can directly reduce the production of lipid ROS [14]. Nuclear factor erythroid 2-related factor 2 (NRF2) is a transcription factor located in the mammal nucleus that protects cells from oxidative stress [15]. Kelch-1ike ECH- associated protein l (KEAP1) inhibits the nuclear translocation of NRF2 and promotes its ubiquitination degradation through NRF2 binding. The KEAP1/NRF2 complex regulates the level of intracellular oxidative stress [16]. Previous studies demonstrated that NRF2 regulates GPX4 and xCT expression by binding to their promoters [17,18].

Micheliolide (MCL), which is derived from parthenolide, is a compound with antioxidant and anti-inflammatory effects and has multiple roles in tumors and inflammatory diseases [19]. ACT001 is a derivate of MCL, MCL/ACT001 is currently in phase II clinical trials for the treatment of glioblastoma (GBM) and has demonstrated *anti*-GBM activity [20,21]. Moreover, previous literature also suggested that MCL could be used as a specific inhibitor of NF-κB to inhibit the expression of inflammatory factors [[22], [23], [24]]. Furthermore, it was also found that MCL protects against doxorubicin-induced cardiotoxicity by regulating NF-κB pathway [25]. However, the effects of MCL on AS progress and macrophage ferroptosis are still unclear.

In the present study, we aimed to investigate the therapeutic effects and molecular mechanisms of MCL on the AS. We found that MCL attenuated AS progression by suppressing macrophage ferroptosis via affecting the formation of KEAP1/NRF2 complex. Our results offered new insights into the mechanisms of MCL in the treatment of AS and provided a therapeutic approach that limit plaque progression by suppressing macrophage ferroptosis via binding to the Arg483 site of KEAP1 with NRF2 competitively.

## 2 Methods

### 2.1 Animals

The study was conducted according to the guidelines of the Declaration of Helsinki and approved by the institutional research ethics committee of the Second Affiliated Hospital of Harbin Medical University (SYDW2019-259, 2019/11/07). All animal experiments were performed in accordance with National Institutes of Health guide for the care and use of laboratory animals. Male ApoE^−/−^mice (6 weeks old) in C57BL/6 background were purchased from Beijing Vital River Laboratory Animal Technology Co., Ltd. (Beijing, China). The ApoE^−/−^ mice had free access to food and water and were housed in the animal center of the Second Affiliated Hospital of Harbin Medical University (22 ± 2 °C, 55 ± 5% relative humidity with a 12-h light/dark cycle).

According to the experimental requirements, ApoE^−/−^ mice were divided into the following groups: normal diet (ND) group: normal chow diet supplemented with equal saline intragastric administration for 16 weeks; high fat diet group: high fat western type diet (21% fat, 0.15% cholesterol; MD12015; Medicine Ltd., Jiangsu, China) supplemented with equal saline intragastric administration; MCL group: according to previous studies [26,27], high fat western type diet supplemented with 100 mg/kg/day MCL intragastric administration. AAV-sh-NC group: the mice were received intravenous injection of adeno-associated virus (AAV) carrying the negative control (NC) plasmids (100 μL vehicle containing 1.0 X 10^12^ AAV2/9 vector particles, one-time injection [12]), Sequence: TTCTCCGAACGTGTCACGTAA. 2 weeks later, the mice were received high fat western type diet supplemented with 100 mg/kg/day MCL intragastric administration; AAV-sh-NRF2 group: the mice were received intravenous injection of AAV carrying the sh-NRF2 plasmids (100 μL vehicle containing 1.0 X 10^12^ AAV2/9 vector particles, one-time injection), Sequence: CTTGAAGTCTTCAGCATGTTA. 2 weeks later, the mice were received high fat western type diet supplemented with 100 mg/kg/day MCL intragastric administration; KEAP1-WT group: the mice were received intravenous injection of AAV carrying the KEAP-WT plasmids(100 μL vehicle containing 1.0 X 10^12^ AAV2/9 vector particles, one-time injection), 2 weeks later, the mice were received high fat western type diet supplemented with 100 mg/kg/day MCL intragastric administration; KEAP1-R483S group: the mice were received intravenous injection of AAV carrying the KEAP-R483S plasmids(100 μL vehicle containing 1.0 X 10^12^ AAV2/9 vector particles, one-time injection), 2 weeks later, the mice were received high fat western type diet supplemented with 100 mg/kg/day MCL intragastric administration; The sequence of KEAP-R483S and KEAP1-WT were shown in Supplemental Table 1. The AAV were purchased from HanBio Tech (Shanghai, China). At the end of 16 weeks, all animals were anesthetized with an intraperitoneal injection of sodium pentobarbital (50 mg/kg body weight) and then euthanized by cervical dislocation. The plasma was collected and frozen immediately. The hearts and aortas were removed carefully and fixed with 4% paraformaldehyde (4 °C for 24 h) or kept in optimal cutting temperature compound (OCT) for immunostaining.

### 2.2 Macrophage culture

THP-1-derived macrophages were cultured in 1640 medium (10% fetal bovine serum, 1% penicillin) and passed every 2–3 days. According to previous literature 25130606, THP-1 was stimulated by 50 nM phorbol myristate acetate (PMA) for 48 h to induce macrophages. Macrophages were pretreated with 10 μM of MCL for 1 h and then treated with 100 μg/ml of ox-LDL for 48 h [28].

### 2.3 Reagents

MCL was purchased from MedChemExpress (New Jersey, USA). Cell Counting Kit (CCK-8) was purchased from Yeasen Biotech Co., Ltd. (Shanghai, China). Ox-LDL was purchased from Yiyuan Biotechnology (Guangzhou, China). SYBR Green and Reverse Transcription kit were obtained from Roche (Basel, Switzerland). Primary antibody sources were listed: antibodies against GPX4 (ab125066) and xCT (ab307601) were purchased from Abcam (Cambridge, UK); KEAP1 (P586) and NRF2 (D1Z9C) were purchased from Cell Signaling Technology (Boston, USA), antibody against β-actin and Goat anti-rabbit antibody were obtained from Merck (Darmstadt, Germany). MDA Assay Kit, GSH assay, ROS Assay Kit, Total Superoxide Dismutase Assay Kit with NBT and LDH Cytotoxicity are obtained from Beyotime Biotechnology (Shanghai, China). Enzyme linked immunosorbent assay (ELISA) kits for TNF-α, IL-1β, IL-10 and IL-4 was obtained from Ebioscience (Wuhan, China). ML385 was obtained from MedChemExpress (New Jersey, USA). Lipid Peroxidation Kit was purchased from Invitrogen (California, USA); Iron Assay Kit was purchased from Leagene (Beijing, China). Adeno associated viral vector (AAV) with sh-NC and sh-NRF2 was purchased from HanBio Tech (Shanghai, China).

### 2.4 Western blot analysis

Total proteins of macrophages and artery were extracted by RIPA (Beyotime Biotechnology, Shanghai, China) containing 1 mM PMSF (Beyotime Biotechnology, Shanghai, China). The BCA kit (Beyotime Biotechnology, Shanghai, China) is used to determine the protein concentration. The samples were added to the sample wells of 10% SDS-PAGE gels for electrophoresis. The proteins are transferred to the PVDF membrane and then sealed with 5% skim milk for 1 h. The PVDF membrane was placed into the primary antibody at 4 °C overnight. TBS-T wash three times. The PVDF membrane was placed into the second antibody for 1h at room temperature. TBS-T wash three times. The protein bands were treated with ECL working solution (Solarbio, Beijing, China) and visualized using a gel imaging system (Tanon, Shanghai, China). The relative expression levels of the target proteins were determined with Image J software.

### 2.5 Real-time reverse transcription-PCR

Trizol was used to extract total RNA from cells and artery according to the manufacturer's instructions (Invitrogen, California, USA). cDNA was synthesized using the Reverse Transcription kit (Roche, Basel, Switzerland), and quantitative polymerase chain reaction (qPCR) was performed using SYBR Green (Roche, Basel, Switzerland). All data were normalized to the mRNA expression level of GAPDH. Gene-specific primers were shown in Supplemental Table 2.

### 2.6 Immunofluorescence

After the cells and vascular tissues were treated, they were fixed with cold acetone for 10 min and washed three times with PBS. And incubated with 5% BSA and 0.3% Triton × 100 for 1h at room temperature. Cells or tissues are added to the primary antibody according to the instructions. After overnight at 4 °C, wash with PBS three times. Fluorescently labeled secondary antibodies were incubated at room temperature for 2 h. Subsequently, DAPI solution was used to dye the nucleus for 5min. After washing the sections three times with PBS. Then, the aortic root tissues were imaged using a fluorescence microscope (Leica, Wetzlar, Germany).

### 2.7 Lipid peroxidation determination

According to previous study [29], when macrophages are treated in different groups, the cells were stained with 10-μM lipid peroxidation sensor for 30min at 37 °C. The lipid peroxidation levels of macrophages were analyzed by flow cytometry.

### 2.8 Transmission electron microscopy

Transmission electron microscopy (TEM) was used to analysis the mitochondrial shape. Briefly, the macrophages were centrifuged at 3,000 rpm/min for 20 min and the supernatant was discarded. The cells were post fixed in cacodylate-buffered 1% osmium tetroxide, dehydrated in ethanol series, and embedded in Poly/Bed 812. Mitochondrial ultra structure was observed in different groups.

### 2.9 MDA level and SOD activity

According to previous study [30], malondialdehyde (MDA) and SOD activity in macrophages and artery lysates were measured with commercial kits (#S0131, #S0101 M, Beyotime) at a wavelength of 532 nm or 450 nm, respectively, following the manufacturer's protocols.

### 2.10 GSH level

GSH level in macrophages and artery lysates was analyzed by glutathione assay kit (Jiancheng, Nanjing, China) following the manufacturer's instruction. After the macrophages are treated, 100 μl of cold PBS was added and the macrophages were collected. 100 μl of precipitant was added into the macrophages. After centrifuged at 3500 rpm for 10 min, supernatant was extracted and mixed with 125 μl working solution for 5min. Luminescence was measured at 405 nm by LMax Microplate Reader (Molecular Devices). GSH concentration in each group was normalized to protein concentration.

### 2.11 CCK8 and LDH assay

After the macrophages were treated in different groups, 10 μl CCK8 working solution was diluted into 100 μl 1640 medium and incubated in 96-well plates for 1 h. Measurement of absorbance at 460 nm. The cell supernatant in different groups was collected and the LDH content was detected according to the manufacturer's instructions (Jiancheng, Nanjing, China).

### 2.12 Iron content assay

The iron levels in plasma and macrophages were detected by Iron Assay Kit (Leagene, Beijing, China). According to the manufacturer's instructions, the levels of iron in blank, iron standard solution and test samples were measured. The content of iron in target group was detected by colorimetry.

### 2.13 HE, masson and oil red staining

Mouse aortic roots were sequentially sliced and subsequently stained with HE (Solarbio, China), Masson (Solarbio, China) and Oil red (Solarbio, China) according to the manufacturer's instructions.

### 2.14 ChIP

The binding of NRF2 to GPX4 and xCT promoter was analyzed using Pierce™ Agarose ChIP Kit (Thermo Fisher). Protein-DNA complexes were stabilized by 4% paraformaldehyde. After ultrasonic fragmentation, protein-DNA complexes were immunoprecipitated by primary antibody to NRF2(1:100, # 12721, Cell Signaling Technologies, Beverly, MA, USA) or rabbit IgG (1:100, #66362, Cell Signaling Technologies). The cross-linked complexes were extracted and solubilized, and the enrichment levels of GPX4 and xCT promoter were analyzed by qPCR.

### 2.15 JC-1 and ferrous ion staining

Macrophages were planted in 48-well plates and treated according to group. Mitochondria membrane potential (JC-1 assay, Beyotime, C2006) and iron ion (ferroOrange, DOJINDO, F374) staining were performed on macrophages according to manufacturer's instructions.

### 2.16 Agilent Seahorse XF cell mito stress test assay

The oxygen consumption rate (OCR), basal respiratory, maximal respiratory and ATP production respiratory were measured with an Agilent Seahorse XFe96 analyzer (Agilent Technologies, Santa Clara, CA, USA). Briefly, 2 × 104 cells/well were seeded in a 96-well XF cell culture microplate in 1640 medium 48 h before assay. The 1640 was replaced with Seahorse medium (pH 7.4) and the cells were incubated at 37 °C for 45 min in incubator. Then the cells were injected following sequential of oligomycin (1.5 μM), FCCP (2 μM), and Rot/AA (0.5 μM). Data were analyzed using the Seahorse XF Cell Mito Stress Test Report Generator package with results normalized against cell number.

### 2.17 Plasmid transfection

The mammalian expression plasmids containing KEAP1-WT, or KEAP1 with single mutations at R483S were purchased from HanBio Tech (Shanghai, China). KEAP1 with single mutations at Q364S and A414S plasmids were kindly provided by Shanghai Institute of Nutrition, Chinese Academy of Sciences. The macrophages were transfected using Lipo3000 reagent (Invitrogen, USA), according to the manufacturer's instructions. Briefly, 2 μl Lipo3000 reagent and 2 μl plasmid (0.8 μg) were diluted into 50 μl Opti-MEM and incubated at room temperature for 5 min before mixing and incubation for a further 20 min.

### 2.18 Knock down of NRF2 expression by siRNA

The siRNA targeting NRF2 were applied to knock down expression of NRF2. The scramble siRNA (Si-NC) and si-NRF2 and obtained by Invitrogen (Carlsbad, CA, USA). After seeding into 6-well plates, macrophages were transfected with 50 nM of Si-NC or Si-NRF2 using Lipofectamine 3000 (Invitrogen). The sequence of NRF2 siRNA: (5′-ATGCAACAGGACATTGAGCAA-3′).

### 2.19 Statistical analysis

All the results were presented as mean ± standard error of mean, and all the data were acquired from at least three independent experiments. Two-tailed Student's t-test were used to compare data between two groups with normal distributed values. Mann-Whitney *U* test was used to compare data between two groups with non-normal distributed values. Statistical analysis was performed by SPSS 21.0 software. P < 0.05 was considered statistically significant.

## 3 Results

### 3.1 MCL administration attenuated the progress of AS in ApoE^−/−^ mice

**Fig. 1:**
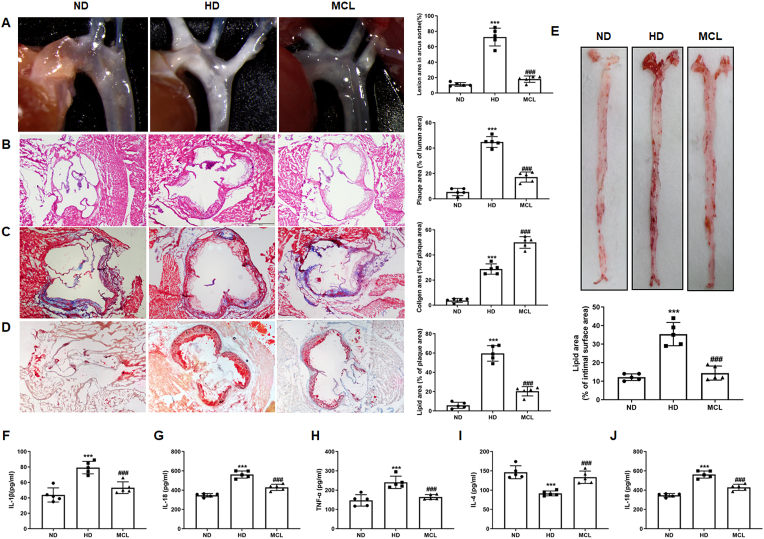
MCL oral administration attenuates the progression of atherosclerosis and improves the inflammatory response in ApoE^−/−^ mice. Animals are divided into: ND group, HD group and HD + MCL group. After the experiment, the tissue is harvested and stained with HE, Masson and Oil red O, the serum is collected to analyze the IL-4, IL-10, IL-18, TNF-α, and IL-1β levels. (A) Gross morphology of mouse aortic arch. N = 5; (B–D). Representative images for HE, Masson, and Oil red O stain in aortic roots. N = 5; (E). Oil red O staining of mouse aortas surface. N = 5; (F). IL-1β level in serum. N = 5; (G). IL-18 level in serum. N = 5; (H). TNF-α level in serum. N = 5; (I). IL-4 level in serum. N = 5; (J). IL-10 level in serum. N = 5. ***p < 0.001 vs ND group; ###p < 0.001 vs HD group.

To investigate whether MCL has a protective effect on AS, ApoE^−/−^ mice were fed western diet supplemented with MCL for 16 weeks. We found the plaque area at the aortic arch and aortic root was significantly reduced in MCL treatment group (Fig. 1A and B). In addition, Masson staining demonstrated that MCL increased the content of collagen in the atherosclerotic plaques (Fig. 1C). Oil red staining for aortic root and artery surface illustrated that, compared with MCL group, the lipid area was diminished in MCL group (Fig. 1D and E). Furthermore, to evaluate the effect of MCL on the inflammatory response in ApoE^−/−^ mice, mice serum inflammatory cytokines level was detected. It was observed that the level of IL-1β, IL-18 and TNF-α, the typical pro-inflammatory factors, were decreased in MCL group (Fig. 1F-H). The levels of the classic anti-inflammatory factors IL-4 and IL-10 were also measured. The results showed that MCL treatment can increase the serum level of IL-4 and IL-10 in ApoE^−/−^ mice (Fig. 1I and J). Moreover, MCL also improved blood lipid levels of the mice. MCL decreased the serum TG, TC and LDL-C level (Supplemental Figs. 1A–C), while increased the HDL-C level (Supplemental Fig. 1D). Taken together, these data suggested that MCL not only attenuated AS development, bult also improved the lipid level and inflammatory response in ApoE^−/−^ mice.

### 3.2 MCL suppressed macrophage ferroptosis and improved oxidative stress in atherosclerotic plaques

**Fig. 2:**
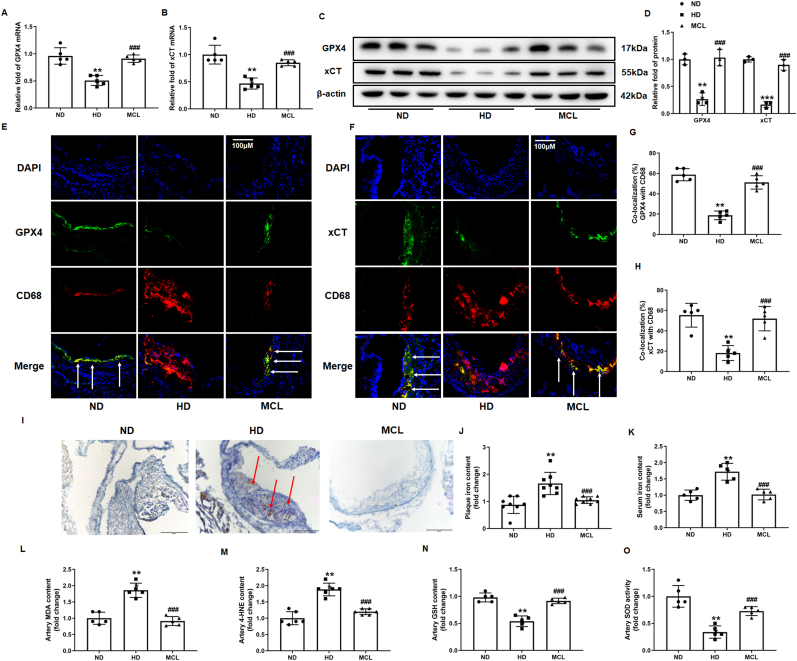
MCL oral administration alleviated the ferroptosis of macrophages in ApoE−/− mice. (A, B). Total artery RNA was extracted from the mice, and mRNA levels of GPX4 and xCT were detected; N = 5. (C, D) Total artery lysates were extracted from the mice, and protein levels of GPX4 and xCT were detected; N = 3. (E, G) GPX4 and CD68 co-staining in aortic sinus. N = 5; (F, H). xCT and CD68 co-staining in aortic sinus. N = 5; (I, J) Representative images and quantification of iron deposition on mouse aortic roots. N = 8; (K) Serum iron content in ApoE^−/−^ mice. N = 5; (L) MDA levels in mouse arterial lysates. N = 5; (M) 4-HNE levels in mouse arterial lysates. N = 5; (N) GSH levels in mouse arterial lysates. N = 5; (O) SOD activity in mouse arterial lysates. N = 5; ***p < 0.001 vs ND group; ###p < 0.001 vs HD group.

Given the vital role of macrophage ferroptosis in the development of AS, we speculate whether MCL might ameliorate AS by inhibiting macrophage ferroptosis. Total RNA from mice artery was extracted to analyze GPX4 and xCT mRNA level, it was found that the mRNA level of GPX4 and xCT were decreased in HD group, while MCL treatment reverted this change (Fig. 2A and B). We further analysis the GPX4 and xCT protein level in total lysates of artery, and found that the result is consistent with the changes of mRNA levels. MCL treatment increased the expression of GPX4 and xCT level in HD group (Fig. 2C and D). Moreover, immunofluorescence was used to observe the level of GPX4 and xCT expression changes in macrophages of atherosclerotic plaque. We found that the expression of GPX4 and xCT level was also decreased in macrophages, while the MCL supplement inhibited these changes (Fig. 2E–H). Furthermore, immunochemical staining showed that MCL significantly reduced iron deposition of plaques and serum iron content in HD group (Fig. 2I–K). Lipid peroxidation is a key characteristic of ferroptosis. MDA and 4-HNE are released after lipid oxidation, MDA and 4-HNE level are considered as biomarkers for ferroptosis. The MDA and 4-HNE level in artery lysates were detected, the results showed that the concentration of MDA and 4-HNE were reduced significantly in the MCL group (Fig. 2L and M). Moreover, MCL also upregulates the levels of SOD and GSH in aorta (Fig. 2N and O). These results suggested that MCL could attenuate the development of AS via suppressing macrophage ferroptosis.

### 3.3 MCL inhibiting macrophage ferroptosis and improved oxidative stress induced by ox-LDL *in vitro*

**Fig. 3:**
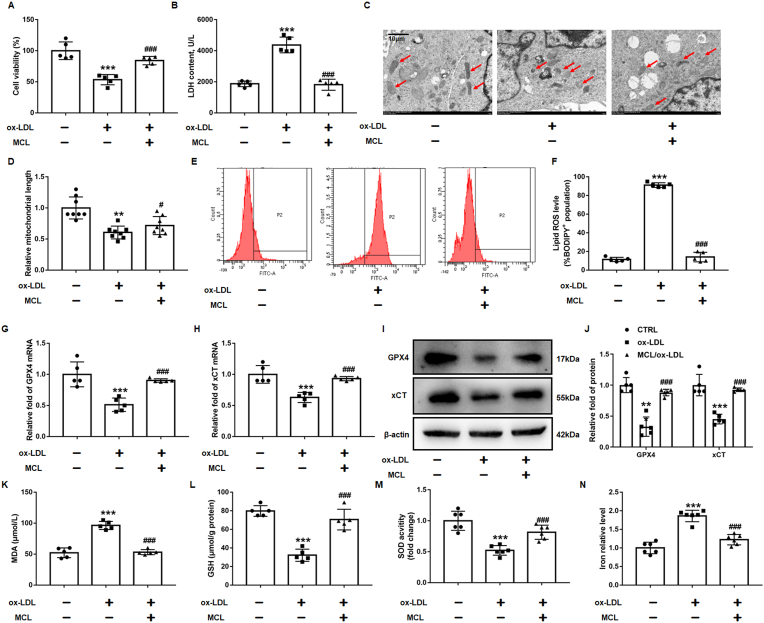
MCL suppresses the ferroptosis of macrophage induced by ox-LDL. THP-1 original macrophages are pretreated with MCL (10 μM) for 1 h, then stimulated with 100 μg/ml ox-LDL for 48 h. (A). CCK-8 assay was used to analysis cell viability. N = 5; (B). LDH level in supernatant. N = 5; (C, D). Representative transmission electron microscopy pictures of macrophages and the quantification of relative mitochondrial length. N = 8; (E, F). The lipid ROS level was evaluated by flow cytometry using C11-Bodipy fluorescent probe. N = 5; (G, H) Total artery RNA was extracted from the cells, and mRNA levels of GPX4 and xCT were detected; N = 5. (I, J) Total cell lysates were extracted, and protein levels of GPX4 and xCT were detected; N = 5. (K–N). The levels of MDA, GSH, SOD activity and iron content were measured in macrophages by commercial kits. **p < 0.01, ***p < 0.001 vs CTRL group; #p < 0.05, ###p < 0.001 vs ox-LDL group.

Previous studies have reported that ox-LDL causes macrophage ferroptosis [31,32]. According to previous studies [31,32], the macrophages were combined with 100 μg/ml ox-LDL for 48 h, we have also found that Fer-1, a specific ferroptosis inhibitor, can improve the decrease of cell viability and lipid peroxidation induced by ox-LDL (Supplemental Figs. 2A and B). The CCK-8 assay was used to evaluate the toxicity of MCL (0, 1.25, 2.5, 5, 10, 20 μM) to macrophages. The results showed that macrophage viability was higher than 90% by using MCL with a concentration below 10 μM (Supplemental Fig. 3). Thus, MCL was administered at a concentration of 10 μM in subsequent experiments. It was observed that 10 μM MCL increased the cell viability and reduced the LDH level in supernatant in ox-LDL treated macrophages (Fig. 3A and B). TEM was used to observe the mitochondrial morphology of macrophages. The results demonstrated that macrophages cultured with ox-LDL had mitochondrial damage, characterized by smaller size and swollen or loss of mitochondria cristae, while MCL pretreatment significantly prevented the ultrastructural changes of mitochondria in macrophages (Fig. 3C and D). We also found MCL improved the lipid ROS level in macrophages (Fig. 3E–F), and the decreased GPX4 and xCT levels in macrophages were revised by MCL treatment (Fig. 3G–J).

**Fig. 4:**
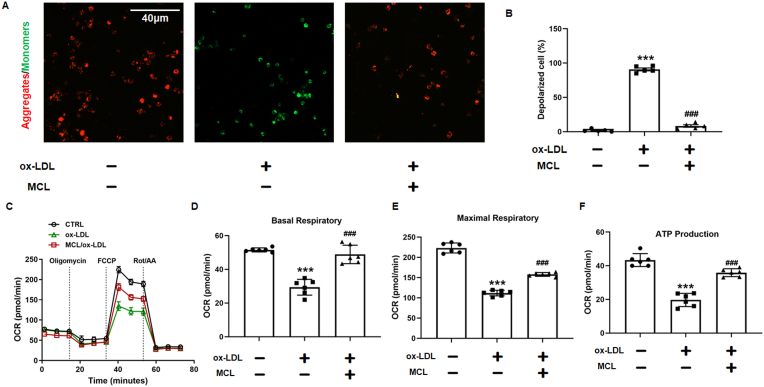
MCL improved macrophage mitochondrial function induced by MCL. THP-1 original macrophages are pretreated with MCL (10 μM) for 1 h, then stimulated with 100 μg/ml ox-LDL for 48 h. (A, B). Representative images for mitochondrial membrane potential. N = 5. (C–F) The mitochondrial function of macrophages was assessed by seahorse assay. N = 5. ***p < 0.001 vs CTRL group; ###p < 0.001 vs ox-LDL group.

Iron excess is the main cause of ferroptosis. According to previous studies, hepcidin/ferroportin (FPN) may be the mechanism leading to excess iron [33,34]. We also found that ox-LDL decreased the FPN and increased hepcidin level, while the MCL reversed these changes (Supplemental Figs. 4A and B). Moreover, *In vivo* experiment also found that MCL increased FPN and decreased hepcidin level (Supplemental Fig4 C-F). Furthermore, MCL decreased MDA level in ox-LDL treated macrophages (Fig. 3K). Consistently, the SOD activity and GSH level were significantly increased in MCL group (Fig. 3L and M). It was also observed that MCL reduces the elevated concentration of iron ions in macrophages induced by ox-LDL (Fig. 3N and Supplemental Figs. 5A and B). Additionally, mitochondrial membrane potential was also measured, and the JC-1 staining showed that MCL significantly improved the loss of membrane potential induced by ox-LDL (Fig. 4A and B). In addition, the results from seahorse shown that MCL can also improve mitochondrial function (Fig. 4C). The Mito Stress Test Assay results revealed significantly increased basal, maximal, and ATP-coupled mitochondrial oxygen consumption rates in ox-LDL-treated cells incubated with MCL compared with the cells only treated with ox-LDL (Fig. 4D–F). To evaluate whether MCL has a saving effect on macrophages after ox-LDL injury, ox-LDL treated macrophages for 24 h and then treated with MCL for 24 h, we found that the protective effect was similar to that of MCL pretreatment (Supplemental Figs. 6A–D). Furthermore, RSL3, a specific ferroptosis inducer, was used to induce macrophage ferroptosis, and we also found that MCL can also inhibit the decrease of macrophage viability and lipid peroxidation (Supplemental Figs. 7A–C). We also evaluated the protective effect of MCL on macrophage pyroptosis and apoptosis, and found that MCL could not inhibit the increased expression of cle-caspase3, cle-caspase9, cle-caspase1 and GSDMD induced by ox-LDL (Supplemental Figs. 8A–D). These data showed that MCL significantly inhibited macrophage ferroptosis and improved mitochondrial function.

### 3.4 MCL enhanced NRF2 nuclear translocation in ox-LDL cultured macrophages

**Fig. 5:**
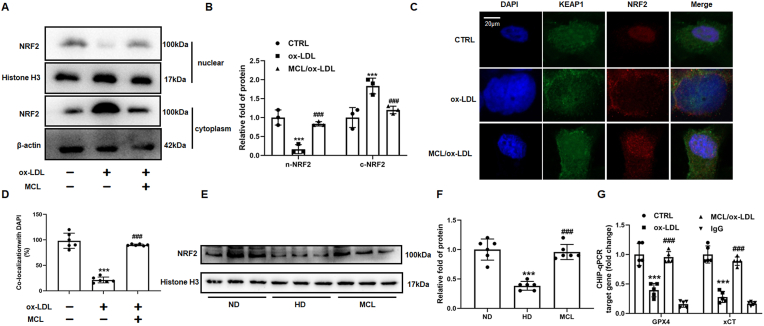
MCL enhances NRF2 nuclear translocation in macrophages and ApoE−/− mice. In macrophages, the nuclear lysate was extracted to analysis NRF2 expression. In macrophages and aorta, Nuclear and cytoplasmic lysate were extracted to analysis NRF2 expression. (A, B). Western blot is used to evaluate NRF2 levels. N = 3; (C, D). Confocal fluorescent staining is used to evaluate the nuclear translocation of NRF2. N = 6; (E, F). The protein levels of NRF2 in the aorta nuclear lysates of ApoE^−/−^ mice. N = 5; (G) The enrichment ability of NRF2 on GPX4 and xCT promoter using ChIP-qPCR; ***p＜0.001, vs control or ND group. ###p < 0.001, vs ox-LDL or HD group.

Previous studies have reported that NRF2 regulates transcription of GPX4 and xCT [29,35,36], we speculate whether MCL might activate NRF2 pathway to increase GPX4 and xCT expression in macrophages. The cytoplasm and nuclear lysates of macrophages were extracted in different treatment groups, respectively. We found that MCL reduced the NRF2 protein expression in the cytoplasm lysates, but significantly increased the protein level of NRF2 in the nucleus lysates (Fig. 5A and B). The results of immunofluorescence were consistent with Western blot (Fig. 5C and D). Moreover, it was also found that MCL reverted the decreased several antioxidant genes regulated by NRF2, such as FTH1, GCLC and NQO1 (Supplemental Fig. 9). Furthermore, to further confirm this result *in vivo*, the nuclear lysate from mouse aorta was isolated. It can be observed that the NRF2 protein level was decreased in HD group, while MCL increased the nuclear NRF2 expression (Fig. 5E and F). Moreover, ChIP-qPCR experiments were performed, we detected that NRF2 significantly enriched the promoter sequence of GPX4 and xCT, and compared to ox-LDL group, the enrichment of the GPX4 and xCT promoter sequence by NRF2 was significantly increased under MCL treatment (Fig. 5G). These data demonstrated that MCL enhanced NRF2 nuclear translocation and increased GPX4 and xCT transcription.

### 3.5 MCL inhibited macrophage ferroptosis and attenuated AS by activating NRF2 pathway

**Fig. 6:**
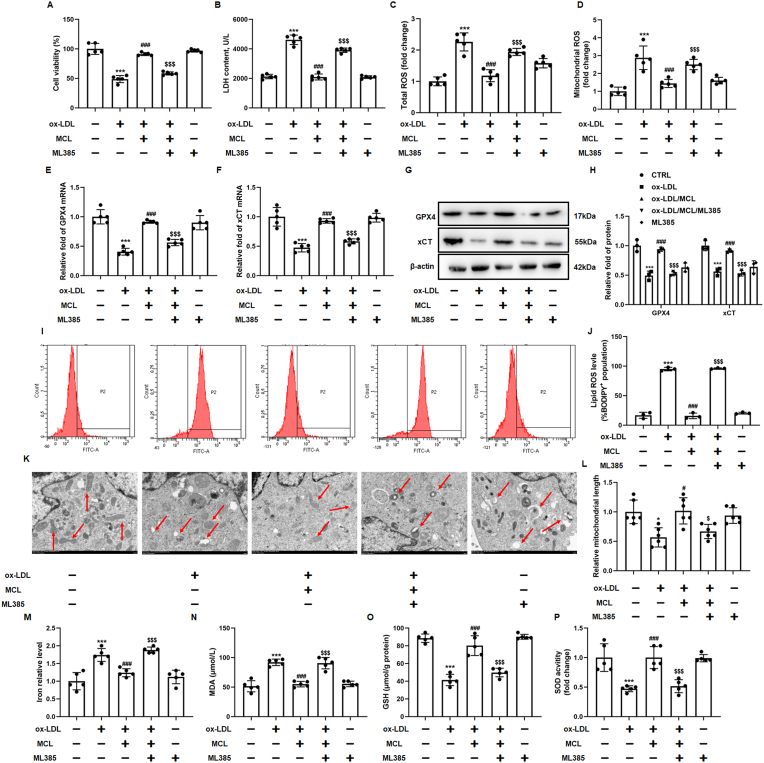
ML385 abolishes the *anti*-ferroptosis and antioxidant effect of MCL. 5 μM ML385 was pretreated in 1 h, then 10 μM MCL is added into the medium, after 1 h, 100 μg/ml ox-LDL is also added into the medium for 48 h. Immunofluorescence is used to observed the ROS staining, microplate reader is used to evaluated the intensity of ROS. The macrophages are divided into five groups, control groups, ox-LDL groups (ox-LDL, 100 μg/ml), MCL group (pretreated with 10 μM MCL for 1h, then combined with 100 μg/ml ox-LDL for 24 h), ML385 group (pretreated with 5 μM ML385 and 10 μM for 1 h, then combined with 100 μg/ml ox-LDL for 48 h), ML385 alone group (5 μM ML385). (A). CCK-8 assay was used to analysis cell viability. N = 5; (B). LDH level in supernatant. N = 5; (C, D) Total and mitochondrial ROS level. N = 5; (E, F) Total artery RNA was extracted from the cells, and mRNA levels of GPX4 and xCT were detected; N = 5. (G, H) Total cell lysates were extracted, and protein levels of GPX4 and xCT were detected; N = 3. (I, J). The lipid ROS level was evaluated by flow cytometry using C11-Bodipy fluorescent probe. N = 3; (K, L). Representative transmission electron microscopy pictures of macrophages and the quantification of relative mitochondrial length. N = 6; (M–P). The levels of MDA, GSH, SOD activity and iron content were measured in macrophages by commercial kits. *p < 0.05, ***p < 0.001 vs control group. #p < 0.05, ###p < 0.001 vs ox-LDL group. $p < 0.05, $$$p < 0.001 vs MCL + ox-LDL group.

To further confirm that MCL inhibits macrophage ferroptosis by activating NRF2 pathway. M385, a specific inhibitor for NRF2, was used to pretreat macrophages. CCK-8 and LDH assay demonstrated that ML385 attenuated the increased macrophage viability and decreased supernatant LDH level in MCL group (Fig. 6A and B). NRF2 controls the transcription and translation of many antioxidant proteins, and compared with MCL group, we also observed a significant increase in total ROS and mitochondrial ROS in the ML385 group (Fig. 6C and D). Similarly, we found that the elevated GPX4 and xCT protein expression after MCL treatment were significantly eliminated by ML385 (Fig. 6E–H). C11 bodipy staining and TEM were used to confirm whether MCL inhibits lipid peroxidation and mitochondrial damage through activating NRF2, the results showed that inhibiting NRF2 by ML385 abolished the protective effects of MCL on the lipid ROS and mitochondrial damage (Fig. 6I-L). Moreover, we found that, compared with MCL group, the iron content and MDA level were also increased after ML385 treatment (Fig. 6M and N). The MCL-induced GSH level and SOD activity were abrogated by ML385 (Fig. 6O and P). Furthermore, Si-NRF2 was used to knock down the level of NRF2 in macrophages and we found that the results were consistent with ML385 (Supplemental Fig. 10A). Si-NRF2 attenuated the effect of MCL on improving cell viability and increasing GPX4/xCT levels (Supplemental Figs. 10B–D). Moreover, Si-NRF2 attenuated the effect of MCL on oxidative stress (Supplemental Figs.10E-H). These data demonstrated that MCL inhibits macrophage ferroptosis by activating NRF2 pathway.

**Fig. 7:**
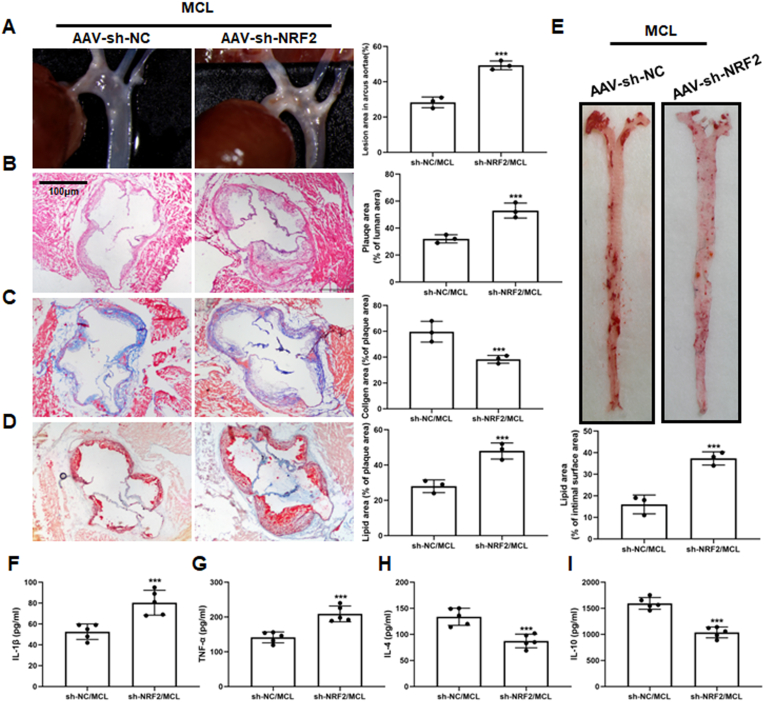
Sh-NRF2 with AAV abolishes the anti-AS effect of MCL in ApoE^−/−^ mice. ApoE^−/−^ mice were divided into two groups and received MCL gavage. The two groups were injected with AAV-sh-NC and AAV-sh-NRF2 via rat tail vein, respectively. After 16 weeks, artery was taken from the mice. (A) Gross morphology of mouse aortic arch. N = 3; (B–D). Representative images for HE, Masson, and Oil red O stain in aortic roots. N = 3; (E). Oil red O staining of mouse aortas surface. N = 3; (F). IL-1β level in serum. N = 5; (G). TNF-α level in serum. N = 5; (H). IL-4 level in serum. N = 5; (I). IL-10 level in serum. N = 5. ***p < 0.001 vs sh-NC/MCL.

Furthermore, to demonstrate that MCL protects AS by activating the NRF2 pathway *in vivo*, we injected ApoE^−/−^ mice with AAV-sh-NRF2 and AAV-sh-NC through the tail vein. It can be observed that in the AAV-sh-NRF2 group, the plaque area at the aortic arch and aortic root was significantly increased (Fig. 7A and B). Masson staining also showed that the increased collogen content induced by MCL was significantly abrogated by AAV-sh-NRF2 injection (Fig. 7C). Furthermore, Oil red staining of aortic roots and artery surfaces also indicated that NRF2 knockdown abolished the inhibitory effect of MCL on lipid deposition (Fig. 7D and E). To confirm whether MCL improves inflammation levels in ApoE^−/−^ mice through activating NRF2, we also analysis serum levels of IL-1β, TNF-α, IL-4 and IL-10. The results showed that NRF2 inhibition can increase the serum IL-1β and TNF-α level, while decrease the IL-4 and IL-10 level in MCL treated mice (Fig. 7F–I). However, knocking down NRF2 did not affect the blood lipid level after MCL treatment (Supplemental Figs. 11A–D). The results illustrated that MCL protects atherosclerosis and improves inflammation response by activating the NRF2 pathway.

### 3.6 MCL inhibited the binding of NFR2 and KEAP1 to promote NRF2 nuclear translocation

**Fig. 8:**
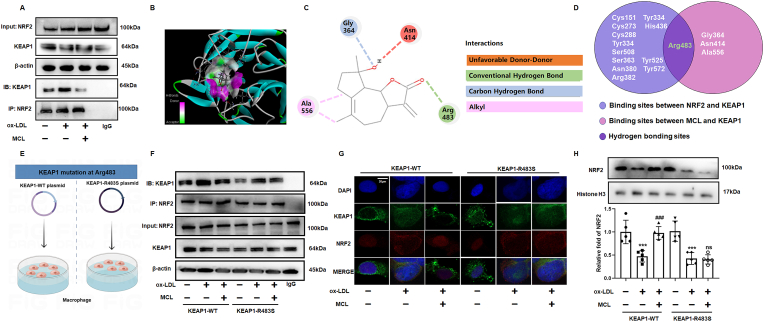
MCL promotes the dissociation of KEAP1 and NRF2 (A). THP-1 original macrophages are pretreated with MCL (10 μM) for 1 h, then stimulated with 100 μg/ml ox-LDL for 48 h. (A). Co-immunoprecipitation was used to evaluate the binding of KEAP1 and NRF2. (B, C). The residues detail of the interaction. (D). Venn diagram representing the overlap of the identified sites between NRF2/KEAP1 and MCL/KEAP1. (E). KEAP1-WT and KEAP1-R483S were transfected into macrophages. (F) Co-immunoprecipitation was used to evaluate the binding of KEAP1 and NRF2. N = 3; (G). Immunofluorescence was used to evaluate the binding of KEAP1 and NRF2. N = 3; (H). In macrophages, the nuclear lysate was extracted to analysis NRF2 expression. N = 5; ***p < 0.001. vs control group. ###p < 0.001 vs ox-LDL group.

NRF2 activity is largely regulated by its interaction with KEAP1. Under physiological conditions, KEAP1 could interact with NRF2 to forms a complex, which inhibits nuclear translocation of NRF2 then results in its degradation by ubiquitination [37]. Under oxidative stress conditions, KEAP1 dissociates from NRF2, which allows NRF2 to enter the nucleus and regulate target genes expression [37,38]. In the present study, we observed that ox-LDL promotes the complex formation of NRF2 and KEAP1, while the effect was inhibited by MCL (Fig. 8A). Previous studies have reported that monomer compounds and proteins can activate the NRF2 pathway by binding with KEAP1 competitively [39,40]. Therefore, we performed molecular docking to predict whether MCL can bind to KEAP1 directly. The results showed that MCL binds to Gly364, Asn414, Arg483 and Ala556 of KEAP1, respectively (Fig. 8B and C). According to the interesting results, we speculate whether MCL promotes the dissociation of NRF2 and KEAP1 by competitively binding KEAP1. Previous studies have identified many amino acid sites on KEAP1 that binds to NRF2 [[41], [42], [43], [44]]. The Wynn diagram showed that the Arg483 amino acid site of KEAP1 was involved in the binding of both KEAP/NRF2 and MCL/KEAP1 (Fig. 8D). The results from molecular docking showed that MCL binds KEAP1 in a hydrogen bond (Fig. 8C), which is also the strongest binding force among these binding sites. To confirm whether MCL competitively binds to Arg483 of KEAP1 to activate NRF2, we transfected the KEAP1 wild type (KEAP1-WT) and Arg483 mutant (KEAP1-R483S, Arg was mutated as Ser) plasmids into macrophages, respectively (Fig. 8E). Immunoprecipitation and immunofluorescence experiments indicated that the effect of MCL on promoting the dissociation of KEAP1 and NRF2 was significantly weakened in KEAP1-R483S mutant macrophages (Fig. 8F and G). Furthermore, we isolated macrophage nuclear lysates and measured the expression of NRF2 levels in different groups, the results showed that KEAP1-R483S mutant abolished the effect of MCL on promoting NRF2 nuclear translocation (Fig. 8H). These data confirmed that MCL promotes KEAP1/NRF2 dissociation and NRF2 nuclear translocation by competitively binding to the Arg483 site of KEAP1.

### 3.7 MCL inhibited macrophage ferroptosis and improved mitochondrial function via binding to the Arg483 site of KEAP1 competitively

**Fig. 9:**
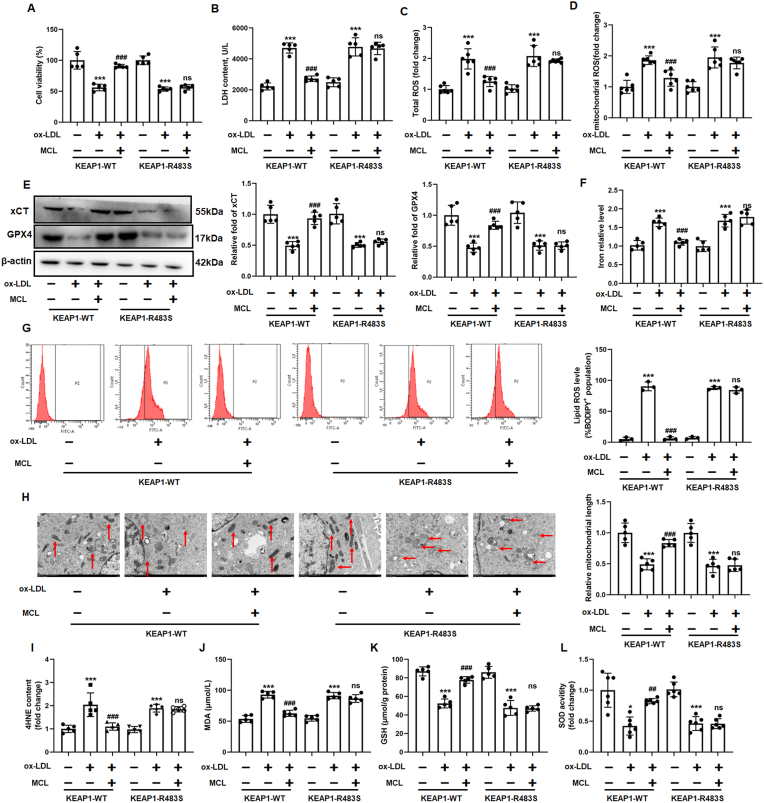
MCL form hydrogen bond with KEAP1 at Arg483 to regulate NRF2 activity and reduce the generation of ROS in ox-LDL treated macrophages. Plasmid with mutated KEAP1 R483S are transfected into macrophages, followed by ox-LDL and MCL treated. (A). CCK-8 assay was used to analysis cell viability. N = 5; (B). LDH level in supernatant. N = 5; (C, D) Total and mitochondrial ROS level. N = 5; (E) Total cell lysates were extracted, and protein levels of GPX4 and xCT were detected. N = 5; (F) The levels of iron content were measured in macrophages by commercial kits. N = 5; (G). The lipid ROS level was evaluated by flow cytometry using C11-Bodipy fluorescent probe. N = 3; (H). Representative transmission electron microscopy pictures of macrophages and the quantification of relative mitochondrial length. N = 5; (I–L) The levels of MDA, GSH, SOD activity and 4-HNE were measured in macrophages by commercial kits. *p < 0.05, ***p < 0.001. vs control group. ##p < 0.01, ###p < 0.001 vs ox-LDL group.

**Fig. 10:**
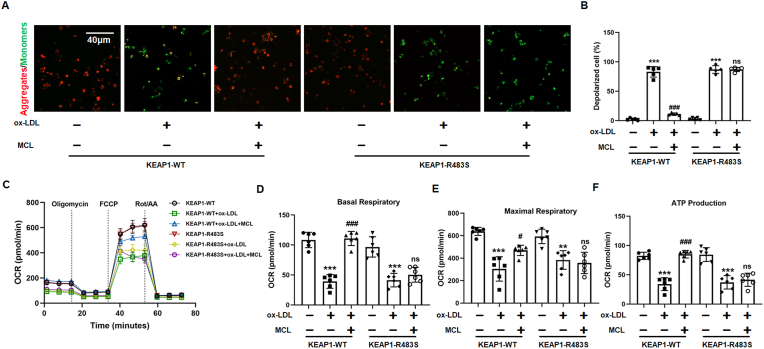
MCL form hydrogen bond with KEAP1 at Arg483 to improve macrophage mitochondrial function induced by ox-LDL. Plasmid with mutated KEAP1 R483S are transfected into macrophages, followed by ox-LDL and MCL treated. (A, B). Representative images for mitochondrial membrane potential. N = 5. (C–F) The mitochondrial function of macrophages was assessed by seahorse assay. N = 5. ***p < 0.001 vs ox-LDL group; ###p < 0.001 vs MCL group.

We further investigated whether MCL inhibit ferroptosis in KEAP1-R483S mutated macrophages. CCK-8 and LDH assay demonstrated that, in KEAP1-R483S mutated macrophages, MCL did not ameliorate ox-LDL-induced decreased cell viability and increased LDH level (Fig. 9A and B). Moreover, the effect of MCL on reducing the total and mitochondrial ROS in KEAP1-R483S mutant macrophages was also eliminated (Fig. 9C and D). Since GPX4 and xCT expression was regulated by NRF2, we examined GPX4 and xCT levels in different treatment of macrophages. It was found that transfection of KEAP1-R483S in macrophages attenuated the effect of MCL on up-regulation of GPX4 and xCT. However, no difference in GPX4 and xCT expression was observed in KEAP1-WT and KEAP1-R483S (Fig. 9E). In addition, MCL reduced iron content and lipid ROS level in KEAP1-WT macrophages, but this effect was eliminated in KEAP1-R483S mutant macrophages (Fig. 9F and G and Supplemental Figs. 12A and B). Moreover, the protective effects of MCL on mitochondrial damage was inhibited in KEAP1-R483S mutated macrophages. We also analyzed the products of lipid peroxidation, the results showed that, in KEAP1-R483S mutated macrophages, MCL cannot inhibit the increased levels of MDA and 4-HNE induced by ox-LDL (Fig. 9I and J). Furthermore, SOD activity and GSH level were significantly improved after MCL treatment in WT plasmid transfected macrophages, but not in KEAP1-R483S transfected macrophages (Fig. 9K and L). JC-1 staining demonstrated that MCL did not improve the loss of cell membrane potential in KEAP1-R483S mutated macrophages induced by ox-LDL (Fig. 10A and B). Moreover, the KEAP1-R483S plasmid transfection alleviated the protection of mitochondrial function by MCL (Fig. 10C). The oxygen consumption analysis also showed that MCL did not increase the basal OCR, maximal OCR, and ATP-coupled OCR in macrophages infected with KEAP1-R483S plasmid (Fig. 10D–F). To further emphasize that MCL inhibits macrophage ferroptosis by binding to Arg483 rather than other amino acid sites, KEAP1-Q364S (KEAP1- Q364S, Gly was mutated as Ser) and KEAP1-A414S (KEAP1- A414S, Asn was mutated as Ser) plasmid were transfected into macrophages. We found that the protective effect of MCL on ferroptosis (Supplemental Figs. 13A–F) and the promotion of NRF2 nuclear translocation (Supplemental Figs. 13G–I) were still observed in mutated macrophages. These results illustrated that MCL suppressed ox-LDL-induced macrophage ferroptosis by binding with the Arg483 site of KEAP1 competitively.

### 3.8 The AS protective effect of MCL was attenuated in ApoE^−/−^ mice with AAV-KEAP1-R483S

**Fig. 11:**
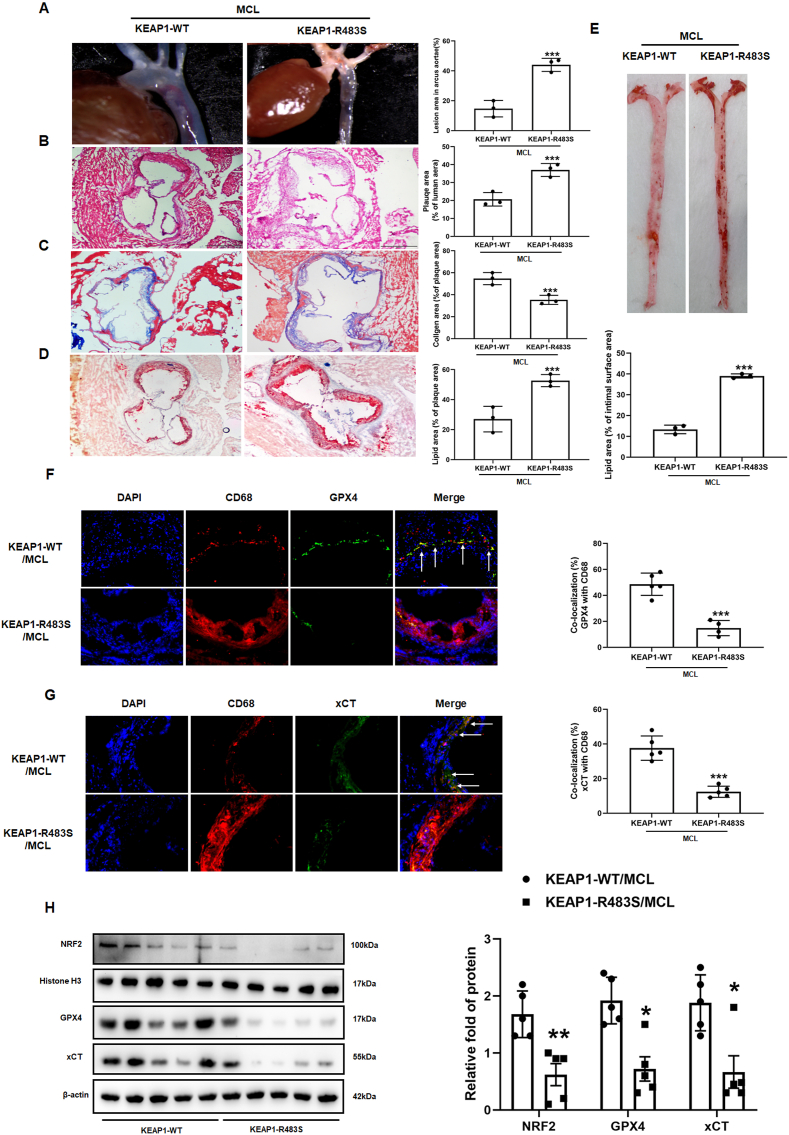
Arg483 mutation weaken the protective effect of MCL on atherosclerosis and macrophage ferroptosis *in vivo*. AAV-KEAP1-WT and AAV-KEAP1-R483S were injected intravenously through the tail of the mouse into 8-weeks-old ApoE^−/−^ mice, all of which were received MCL gavage. (A). Gross morphology of mouse aortic arch. N = 3; (B–D) Representative images for HE, Masson, and Oil red O stain. N = 3; (E). Oil red O staining of mouse aortas surface. N = 3; (F) GPX4 and CD68 co-staining in aortic sinus. N = 5; (G). xCT and CD68 co-staining in aortic sinus. N = 5; (H) Western blot is used to evaluate GPX4, xCT, and nuclear NRF2 levels (N = 5). *p < 0.05, ***p < 0.001. vs KEAP1-WT group.

To further confirm the protective effect of MCL on AS via binding to Arg483 of KEAP1 competitively, AAV with KEAP1-WT and KEAP1-R483S were injected into 8 weeks ApoE^−/−^ mice via tail vein. The mice were fed with HD supplemented with MCL for 16 weeks. Serum lipid level was measured, and we found that the KEAP1-R483S mutation did not affect the effect of MCL on lowering serum lipid levels in the mice (Supplemental Figs. 14A–D). However, the anti-inflammatory effects of MCL were inhibited in KEAP1-R483S mutant mice. Compared with KEAP1-WT mice, the serum levels of IL-1β and TNF-α were significantly increased, while the levels of IL-4 and IL-10 were significantly decreased in KEAP1-R483S mutated mice (Supplemental Figs. 14E–H). The level of MDA and 4HNE were increased, while GSH content and SOD activity were also decreased in KEAP1-R483S mutant mice (Supplemental Fig. 14I-L). The area of plaque at the aortic arch and aortic root was significantly higher in KEAP1-R483S mutated mice (Fig. 11A and B). Masson staining displayed that the content of collagen fiber decreased significantly in KEAP1-R483S mice (Fig. 11C). Oil red staining of the aortic root and aortic surface also found that the effect of MCL on improving lipid deposition was attenuated in KEAP1-R483S mutated mice (Fig. 11D and E). Furthermore, we performed immunofluorescence staining to evaluate GPX4 and xCT levels in macrophages. It can be observed that the colocalization area of CD68, GPX4 and xCT were significantly decreased in KEAP1-R483S mutated mice (Fig. 11F and G). Moreover, Western blot was used to analyze the expression levels of GPX4, xCT and NRF2 in total lysate of artery, aortic homogenate and nuclear homogenate, respectively. The results demonstrated that the effect of MCL on GPX4, xCT and NRF2 expression in the nucleus was significantly inhibited in KEAP1-R483S mutant mice (Fig. 11H). These results revealed that MCL inhibits macrophage ferroptosis and AS progression through competitive binding to Arg483 site of KEAP1.

## 4 Discussion

AS is one of the main pathological processes of the most common cardiovascular diseases. Although the lipid-lowering and anti-inflammatory treatments for AS get great progress, a large proportion of patients still develop as acute myocardial infarction. Therefore, it is urgent to explore a new therapeutic targets and agents for AS. In the present study, the protective effects and underlying mechanisms of a novel small molecule compound MCL against AS were investigated. The main findings of this study are as follows: 1) MCL administration attenuated AS development, decreased inflammatory response and improved oxidative stress in ApoE^−/−^ mice; 2) MCL inhibited macrophage ferroptosis and improved mitochondrial function to attenuate the progress of AS; 3) MCL suppressed macrophage ferroptosis by increasing GPX4 and xCT transcriptional activity via activating NRF2 pathway; 4）MCL promoted KEAP1/NRF2 dissociation by competing with NRF2 to bind the Arg483 site of KEAP1, which resulted in NRF2 translocation to the nucleus. Our present study identifies a potential therapeutic agent for AS and clarifies the specific mechanism by which MCL inhibits AS.

MCL is derived from an ancient anti-inflammatory drug. In recent years, the biological roles and clinical applications of MCL have received extensive attention. There are increasing evidence have confirmed that MCL has anti-tumor and anti-inflammatory effects [24,45,46]. It was shown that MCL cross the blood-brain barrier, and Phase II clinical trials have confirmed that MCL has considerable therapeutic value for glioma [21]. Furthermore, previous studies have shown MCL inhibits inflammation response, suggesting MCL may have a potential role in preventing chronic inflammatory diseases [22,47]. In the present study, we reported that MCL attenuated the development of AS effectively. Mechanistically, we found that MCL suppressed AS by inhibiting macrophage ferroptosis via activating NRF2 pathway. Our study confirms the AS protective effect of MCL and explains the mechanism by which MCL inhibits AS. Furthermore, MCL can reduce plasma levels of IL-I-β, TNF-α, LDL-C, TC, and TG, and increase levels of IL-4, IL-10, and HDL-C in ApoE^−/−^ mice. These data suggested that MCL has potent anti-inflammatory and lipid-improving effects. However, in the present study, only 100 mg/kg MCL was used in ApoE^−/−^ mice, which is the limitation of the present study, and more concentrations should be applied in our subsequent study.

Macrophages plays an important role in the progression of AS [2,48]. In the advanced stage of AS, macrophages are featured by efferocytosis dysfunction, death and increased inflammatory response, which increased the risk of atherosclerotic plaque rupture. Previous studies have reported that macrophage death as could a pharmacological target for the treatment of AS [49,50]. It was reported that GSDME-mediated pyroptosis promotes the AS progression and aggravates the inflammatory response in the plaques [4]. Moreover, previous study found that clonal haematopoiesis aggravates the progression of AS by promoting the AMI2-inflammasome activation and enhanced macrophage pyroptosis [51]. Additionally, our previous study has also confirmed that hyper-homocysteinemia caused macrophage pyroptosis to promote AS development [52]. Furthermore, inhibition of macrophage death has been proved to suppressed AS [39,53]. In the study, we reported that MCL inhibited AS progression by decreasing plaque area, reducing lipid deposition and increasing collagen content in the plaques. Moreover, MCL improved blood lipid levels and decreased the serum pro-inflammatory factors levels. *In vivo* and *in vitro* experimental also demonstrated that MCL inhibited ferroptosis in ox-LDL treated and atherosclerotic plaque macrophages. Therefore, we speculate that inhibition of macrophages ferroptosis is one of the underlying mechanisms of MCL to attenuate AS progress. Macrophage death can lead to enhanced local and systemic inflammatory responses. We also speculate that MCL may also reduce the expression of plasma inflammatory factors by inhibiting macrophage ferroptosis. Previous study has reported that MCL/ACT001 suppressed M1 macrophages polarization via inhibiting P65 pathway [54]. Studies have shown that M1 macrophages contributes to AS progression [55,56]. Therefore, inhibition of macrophage M1 polarization may also be the mechanism by which MCL improves AS. Activation of TLR4/P65 pathway has been confirmed to promote the progression of AS by triggering inflammatory response. Previous studies have confirmed that MCL/ACT001 can inhibit the activation of TLR4/P65 pathway [54,57]. Our data also showed that MCL reduced serum levels of inflammatory factors. Furthermore, we also found that MCL inhibits nuclear translocation and phosphorylation of P65 induced by ox-LDL (Supplemental Figs. 15A–D). Therefore, we speculate that MCL may limit AS progression by inhibiting TLR4/P65 pathway and inflammatory response.

Ferroptosis is a newly discovered programmed cell death, which plays an important role in the development of AS [58,59]. Iron metabolism disorder was regarded as the main cause of ferroptosis. It was reported that circulating ferritin levels were independently correlated with the changes in carotid intima-media thickness [60]. In addition, it has also shown that GPX4 mutations enhance the risks for aortoiliac occlusive disease and peripheral arterial disease that result in atherosclerotic occlusions [61]. These data from patients hinted that ferroptosis may plays an essential role in the progression of AS. Furthermore, it was reported that inhibition of endothelial ferroptosis attenuated the progression of AS [11]. Our previous data also revealed that endothelial cell ferroptosis was observed in estrogen-deficient ApoE^−/−^ female mice, while estrogen supplementation attenuated atherosclerosis by inhibiting endothelial cell ferroptosis [29]. Our previous study has reported that smoking increases plaque instability and the risk of plaque rupture by promoting macrophage ferroptosis [12]. These findings all suggest that ferroptosis may be a potential target for AS treatment. The currently recognized ferroptosis inhibitor is Fer-1, however its mechanism and biological safety remain unclear [62,63]. In the present study, we found that a natural monomer compound, MCL, inhibits ox-LDL and RSL3 induced ferroptosis in macrophages. Furthermore, MCL also increased the expression of GPX4 and xCT in macrophages of atherosclerotic plaques and decreased the levels of MDA and 4-HNE in artery lysates. Iron excess is the main cause of ferroptosis, which triggers the Fenton reaction and results in ROS generation. It was reported that hepcidin/FPN axis contributes to macrophage iron excess induced by ox-LDL. ox-LDL decreased the FPN level by increasing hepcidin expression, which results in an obstruction of iron expulsion from the cell [34]. We found that MCL increased the FPN level and decreased the hepcidin expression. Moreover, this change was also confirmed by *in vivo* experiment. We speculate that MCL may promote intracellular iron transport by increasing the expression of PFN. In atherosclerotic plaques, our group also found that cigarette tar causes iron excess through decreasing FPN level, which ultimately promotes iron death in macrophages [12]. However, the underlying mechanism of MCL inhibiting iron excess still needs further study. We also found that 10 μM MCL cannot inhibit ox-LDL-induced macrophage apoptosis and pyroptosis. The specificity of MCL for ferroptosis protection needs to be further determined, and different concentrations of MCL should be used to evaluate the protective effect. In addition, MCL has demonstrated anti-glioblastoma activity in clinical trials [64]. These data confirm the safety of MCL as a therapeutic agent. Taken together, our data support ferroptosis could be a potential target for the prevention and treatment of AS, and we have identified MCL, which is an effective and safety agent that inhibit AS by inhibiting macrophage ferroptosis.

There is increasing evidence that excessive oxidative stress induced lipid peroxidation is primarily responsible for the progression of ferroptosis [[65], [66], [67]]. NRF2 is a classical transcription factor that regulates the transcription of many antioxidant proteins to maintain redox homeostasis [15,68]. Previous studies have reported that NRF2 plays a critical role in mitigating lipid peroxidation and inhibiting ferroptosis [[69], [70], [71]]. We found that MCL not only promoted NRF2 nuclear translocation, but also increased the expression of GPX4 and xCT. These results confirmed that MCL activate NRF2 activity in ox-LDL treated macrophages. Furthermore, it was also observed that ML385, a specific antagonist of NRF2, abolished the protective effect of MCL on macrophage ferroptosis. Furthermore, *in vivo* data also demonstrated that NRF2 knockdown significantly eliminated the protection effects of MCL. Previous studies have also reported the potential biological effects of MCL on oxidative stress [19,24]. We also observed that MCL reduced total and mitochondrial ROS level, increased the SOD activity and decreased GSH level in ox-LDL injured macrophages. Consistently, ML385 also reverses above-mentioned changes. Therefore, we hypothesize that MCL inhibits oxidative stress, macrophage ferroptosis, and atherosclerosis progression by activating NRF2 pathway. Interestingly, unlike ox-LDL, ML385 increased cell ROS levels but did not affect macrophage viability. In the current manuscript, 5 μM ML385 was used to pretreat macrophages; according to previous literature [29,72], this concentration only inhibited NRF2 activity but did not damage the cells. Our data also explain the underlying mechanism by which we and previous literature have reported the antioxidant effects of MCL. However, previous study reported that even in GPX4-knocked out cells, overexpression of NRF2 can promote ferroptosis resistance in A549 cells [73]. Kim et al. also found that knocking down NRF2 did not decrease GPX4 expression in A549 cells [74]. These results suggested that GPX4 expression is not fully regulated by NRF2 activity, and NRF2 may inhibit ferroptosis independently of GPX4. In the present study, we found ox-LDL decreased nuclear NRF2 and GPX4 level, while MCL reversed the change. Moreover, the results from CHIP assay demonstrated that MCL promotes the binding of NRF2 to the GPX4 promoter, which strongly supports the regulation of GPX4 transcription by NRF2. Similar results have been reported in the previous literature [18,75]. There are several potential reasons, Firstly, NRF2 can regulate the transcription of many antioxidant proteins, and NRF2 may inhibit ferroptosis by increasing the expression of other proteins, such as xCT [76,77]. We also found that MCL enhanced NRF2 nuclear translocation and increased xCT expression. Secondly, GPX4 levels may be regulated by multiple transcription factors, such as NRF1 [73]. NRF2 knockout may lead to the enhancement of NRF1 activity and increase GPX4 expression. Thirdly, the basal expression of NRF2 in A549 cells is very high, which may be the potential cause of this difference [18].

Recently, since the potential cardiovascular-protective effects of NRF2 activation, the mechanism of NRF2 activation has been widely concerned. KEAP1 is considered to be one of the most essential proteins in regulating NRF2 activity [78]. Under different conditions, KEAP1 regulates NRF2 activity by binding or dissociating with NRF2, which maintain intracellular redox homeostasis [79]. Therefore, the amino acid site where KEAP1 binds to NRF2 may be a key target for regulating NRF2 activity. Ji et al. reported that Keap1 S-sulfhydration at Cys151 site induced by hydrogen sulfide suppressed AS progress and liver injury [80,81]. In the present study, we have also previously reported that quercetin promotes the dissociation of KEAP1 and NRF2 and activates NRF2 by binding to KEAP1 [39]. The results of co-immunoprecipitation and immunofluorescence showed that MCL promoted the dissociation of KEAP1 and NRF2. Moreover, we identified potential binding sites for KEAP1 and MCL through molecular docking. However, only Arg483 in these sites is involved in the formation of the KEAP1/NRF2 and KEAP1/MCL complex simultaneously. Therefore, we speculate whether Arg483 site of KEAP1 is a key target for MCL to inhibit macrophage ferroptosis and protect against AS. To confirm this hypothesis, the Arg483 mutant plasmid was transfected into macrophages and injected into ApoE^−/−^ mice, respectively. Rescue experiment demonstrated that the anti-oxidative stress and *anti*-ferroptosis effects of MCL were abolished in R483S mutated macrophages. Moreover, in KEAP1-Q364S and KEAP1-A414S mutated macrophages, MCL can still significantly inhibit ferroptosis and enhance NRF2 nuclear translocation. These *in vitro* data further emphasize that MCL increases NRF2 nuclear translocation and inhibits macrophage ferroptosis by binding to the Arg483 site, but not other sites. Furthermore, *in vivo* experiment showed that, the atherosclerosis and ferroptosis protective effects of MCL were eliminated in ApoE^−/−^ mice with KEAP1-R483S injection. These results demonstrated that the binding of MCL to the Arg483 site of KEAP1 is a key mechanism for its anti-atherogenic effects. The Arg483 site of KEAP1 has also been reported to regulate the activation of NRF2. Lo et al. reported that KEAP1-R483A mutant make KEAP1 unable to bind to NRF2 and repress NRF2 activity in 293T cell lines [82]. Interestingly, we found that KEAP1-R483S mutated macrophages only attenuated the role of MCL in activating NRF2 activity. However, compared with the wild group, the Arg483 site of mutant KEAP1 did not inhibit NRF2 nuclear translocation and the expression of downstream protein. There could be several potential reasons. Firstly, *trans*-infection of KEAP-R483S can only reduce the binding force of KEAP1/NRF2, but KEAP1-R483S can still bind NRF2. This has been reported in previous literature [39]. Secondly, in the present study, endogenous wild KEAP1 is still present in macrophages, which can control the activity of NRF2. Thirdly, it has been reported that in neuron cells, the mutation of Arg415 site on KEAP1 does not affect the binding of KEAP1 and NRF2, but still abolish the role of formononetin in stimulating NRF2 activity [83]. This is consistent with our results, which revealed that the control of NRF2 activity by KEAP1 mutation is also different in different cell lines. These data also suggest that the Arg483 site of mutated KEAP1 in macrophages does not directly promote the dissociation of KEAP1 and NRF2, but only inhibits the role of MCL in activating NRF2 activity. The potential mechanism by which KEAP1-R483S binds to NRF2 and represses NRF2 activity required further investigation.

## 5 Conclusion

In the present study, we report for the first time the protective effect of MCL on AS and partially explain the specific mechanism by which MCL inhibits AS. MCL suppressed atherosclerosis by inhibiting macrophage ferroptosis by improving oxidative stress, and the related mechanism is to promote NRF2 activation through binding to the Arg483 site of KEAP1 competitively. Therefore, our study improves the understanding of the role of MCL on protecting AS and may contribute to expand the cardiovascular clinical application of MCL.

## Funding

This work was supported by Key Research and Development Plan of Heilongjiang Science and Technology Department (SC2022ZX06C0072), the National Natural Science Foundation of China (No. 82370464, 82061130223, No. 82072031, No. 82200454), HMU Marshal Initiative Funding (HMUMIF-21016), Natural Science Foundation of Heilongjiang Province (LH2020H112) and Fund of Key Laboratory of Myocardial Ischemia, Ministry of Education (KF202313, KF202308).

## Consent for publication

Yes.

## Availability of data and material

The data underlying this article will be shared on reasonable request to the corresponding author.

## Authors' contributions

H. J.: Acquisition and drafting the work. B. X., X. L., Y. W, and J. X.: Acquisition of data for the work, X. L., X. Z., and X. B. and Y. C.: Analysis and interpretation of data, X. W., X. L., X. Z., Y. L. and S. Z.: Drafting the article. S.H., J.L., D. Y., Y. T., X. L. and J. D.: Revised the manuscript. H. J.: Acquisition. All the authors approved the final manuscript.

## Declaration of competing interest

The authors declare that there is no conflict of interest in this study.

## Data Availability

Data will be made available on request.
