# Corrigendum to “MCL attenuates atherosclerosis by suppressing macrophage ferroptosis via targeting KEAP1/NRF2 interaction” [Redox Biol. 69 (2024) 102987]

**DOI:** 10.1016/j.redox.2023.103009

**Published:** 2024-01-03

**Authors:** Xing Luo, Yuehong Wang, Xinxin Zhu, Yuwu Chen, Biyi Xu, Xiaoxuan Bai, Xiuzhu Weng, Jinmei Xu, Yangyang Tao, Dan Yang, Jie Du, Ying Lv, Shan Zhang, Sining Hu, Ji Li, Haibo Jia

**Affiliations:** a Department of Cardiology, 2nd Affiliated Hospital of Harbin Medical University, Harbin 150001, PR China; b National Key Laboratory of Frigid Zone Cardiovascular Diseases, The Key Laboratory of Myocardial Ischemia, Chinese Ministry of Education, Harbin, 150001, PR China; c State Key Laboratory of Systems Medicine for Cancer, Division of Cardiology, Renji Hospital, School of Medicine, Shanghai Jiao Tong University, Cancer Institute, Shanghai, 200127, PR China; d Department of Endocrinology, Fourth Affiliated Hospital of Harbin Medical University, Harbin, Heilongjiang, 150001, PR China; e Department of Ultrasound, 2nd Affiliated Hospital of Harbin Medical University, Harbin, 150001, PR China; f Department of Forensic Medicine, Harbin Medical University, Harbin, 150001, PR China

The authors realized that the quantization diagram of the present manuscript Fig. 1J and supplemental Fig. 15D had been incorrectly placed, and Fig. 7E had been incorrectly labeled. Therefore, we request a correction to Fig. 1J, supplemental Fig. 15D and Fig. 7E in order to avoid misunderstanding of the reader. All authors agree on this correction. We promise that this error has no impact on the integrity of the entire article.

**Fig. 1:**
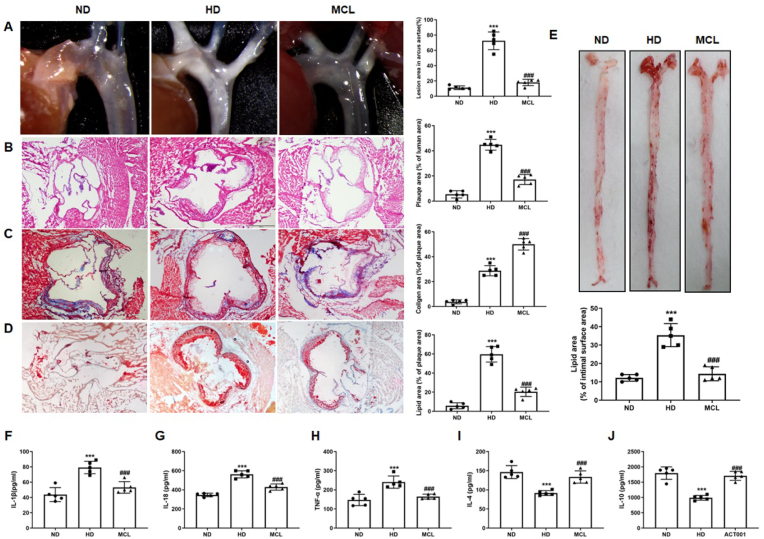
**MCL oral administration attenuates the progression of atherosclerosis and improves the inflammatory response in ApoE**^**−/−**^**mice.** Animals are divided into: ND group, HD group and HD + MCL group. After the experiment, the tissue is harvested and stained with HE, Masson and Oil red O, the serum is collected to analyze the IL-4, IL-10, IL-18, TNF-α, and IL-1β levels. (A) Gross morphology of mouse aortic arch. N = 5; (B-D). Representative images for HE, Masson, and Oil red O stain in aortic roots. N = 5; (E). Oil red O staining of mouse aortas surface. N = 5; (F). IL-1β level in serum. N = 5; (G). IL-18 level in serum. N = 5; (H). TNF-α level in serum. N = 5; (I). IL-4 level in serum. N = 5; (J). IL-10 level in serum. N = 5. ***p < 0.001 vs ND group; ###p < 0.001 vs HD group.

**Fig. 7:**
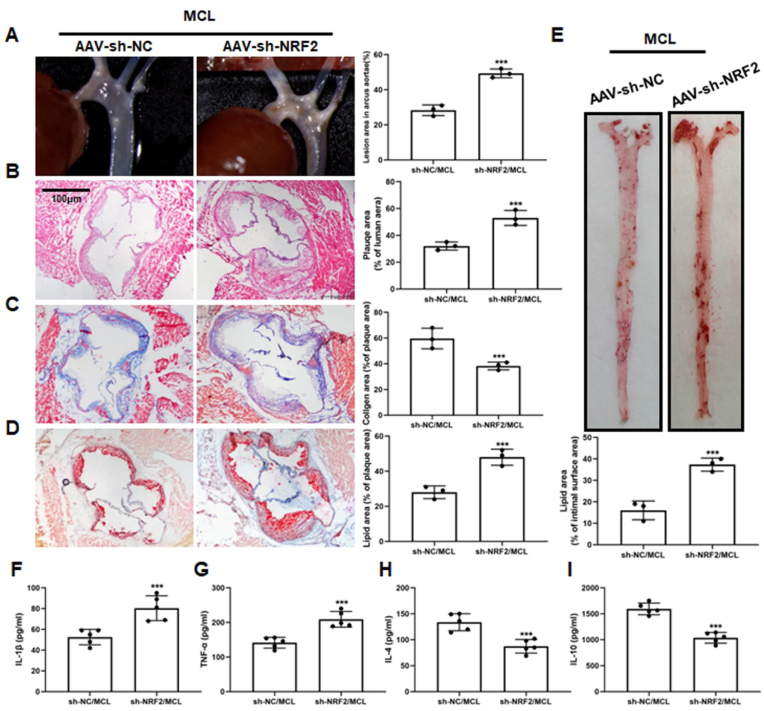
**Sh-NRF2 with AAV abolishes the anti-AS effect of MCL in ApoE**^**−/−**^**mice.** ApoE^−/−^ mice were divided into two groups and received MCL gavage. The two groups were injected with AAV-sh-NC and AAV-sh-NRF2 via rat tail vein, respectively. After 16 weeks, artery was taken from the mice. (A) Gross morphology of mouse aortic arch. N = 3; (B-D). Representative images for HE, Masson, and Oil red O stain in aortic roots. N = 3; (E). Oil red O staining of mouse aortas surface. N = 3; (F). IL-1β level in serum. N = 5; (G). TNF-α level in serum. N = 5; (H). IL-4 level in serum. N = 5; (I). IL-10 level in serum. N = 5. ***p < 0.001 vs sh-NC/MCL.

**Supplemental Fig. 15:**
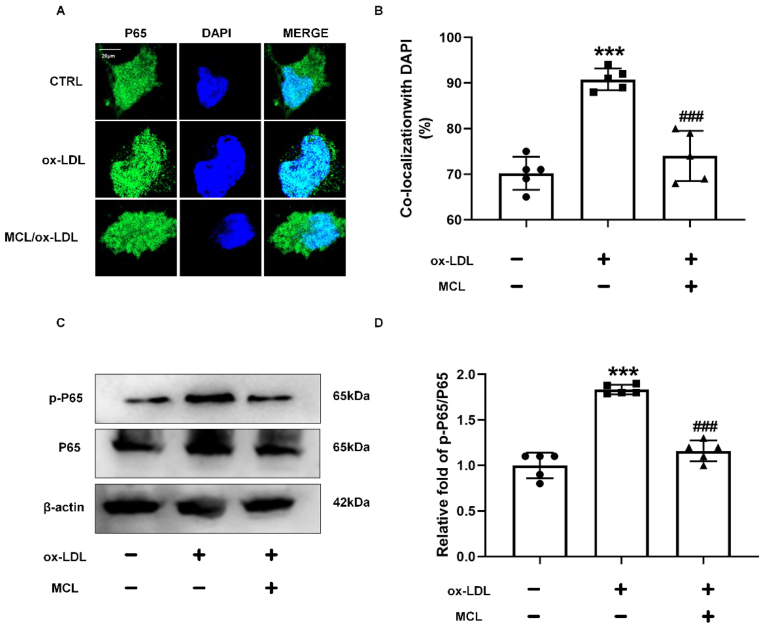
**MCL reverses ox-LDL induced nuclear translocation and phosphorylation of P65.** Macrophages were pretreated with MCL (10 μM) for 1 hour, then treated with ox-LDL (100 μg/ml) for 48 h. (A, B) Confocal fluorescent staining is used to evaluate the nuclear translocation of NRF2. N = 5; (C, D) Western blot is used to evaluate the level of p-P65. ***p < 0.001 vs CTRL group; ###p < 0.001 vs ox-LDL group.

Figure legends

Figure 5(E, F) The protein levels of NRF2 in the aorta nuclear lysates of ApoE mice. N = 6;

Supplemental Fig 8. MCL cannot reduce cle-caspase3, cle-caspase9, cle-caspase1 and GSDMD level in macrophages induced by ox-LDL Macrophages were pretreated with MCL (10 μM) for 1 hour, then treated with ox-LDL (100 μg/ml) for 48 h. (A-C) Western blot is used to evaluate the level of cle-caspase3 and cle-caspase9.; (D-F) Western blot is used to evaluate the level of cle-caspase1 and GSDMD. ***p < 0.001 vs CTRL group;

The authors would like to apologise for any inconvenience caused.

