# Supplementary material for: MCL attenuates atherosclerosis by suppressing macrophage ferroptosis via targeting KEAP1/NRF2 interaction
Source: Redox Biol. 2023 Dec 7;69:102987. doi: 10.1016/j.redox.2023.102987 (PMC10761782; doi:10.1016/j.redox.2023.102987)
Supplement: Multimedia component 1 [file mmc1.docx]

**
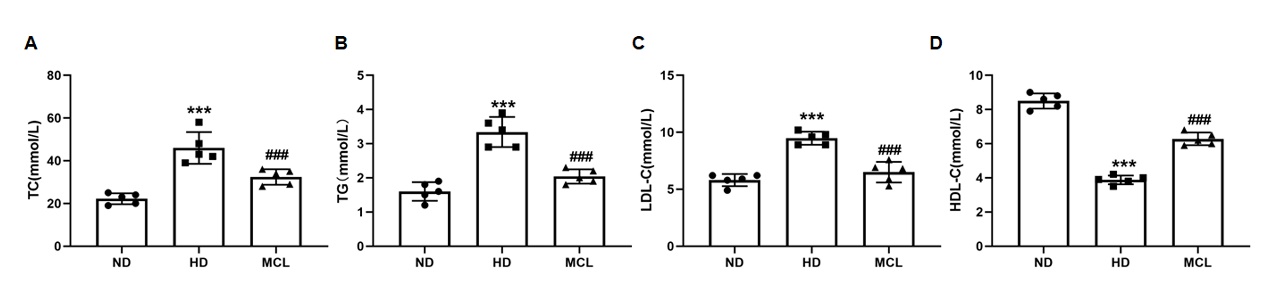
**

**Supplemental Fig. 1. MCL administration improved lipid level in ApoE^-/-^ mice.**

Blood lipid levels in the mice were measured. (A) TC level. N=5; (B) TG level. N=5; (C) LDL-C level. N=5; (D) HDL-C level. N=5; ***p < 0.001 vs ND group; ###p < 0.001 vs MCL group.

**
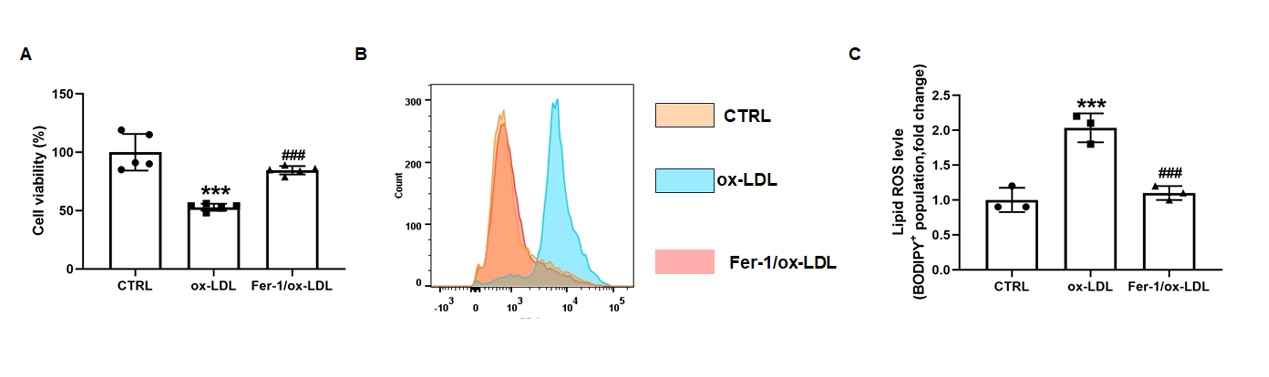
**

**Supplemental Fig 2. Fer-1 inhibited the decrease of cell viability in macrophages induced by ox-LDL**

Macrophages were pretreated with Fer-1 (5 μM) for 1 hour, then treated with ox-LDL (100 μg/ml) for 48 h. (A). CCK-8 assay was used to analysis cell viability. N=5; (B, C). The lipid ROS level was evaluated by flow cytometry using C11-Bodipy fluorescent probe. N=3; ***p < 0.001 vs CTRL group; ###p < 0.001 vs ox-LDL group.

**
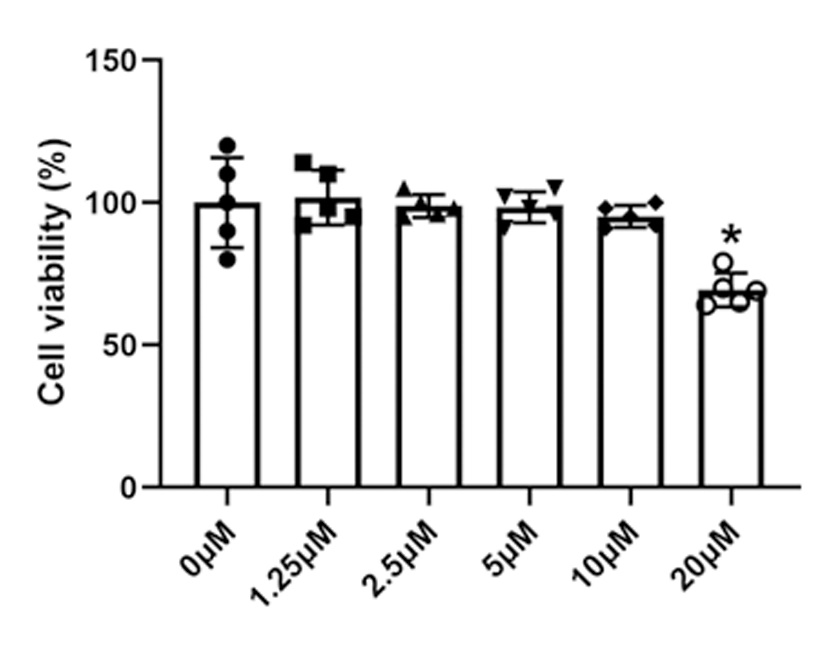
**

**Supplemental Fig. 3. The toxicity of MCL to macrophages**

Macrophages were treated with different concentrations of MCL for 48 hours, and CCK-8 was used to evaluate cell viability. * p < 0.05 vs 0μM.


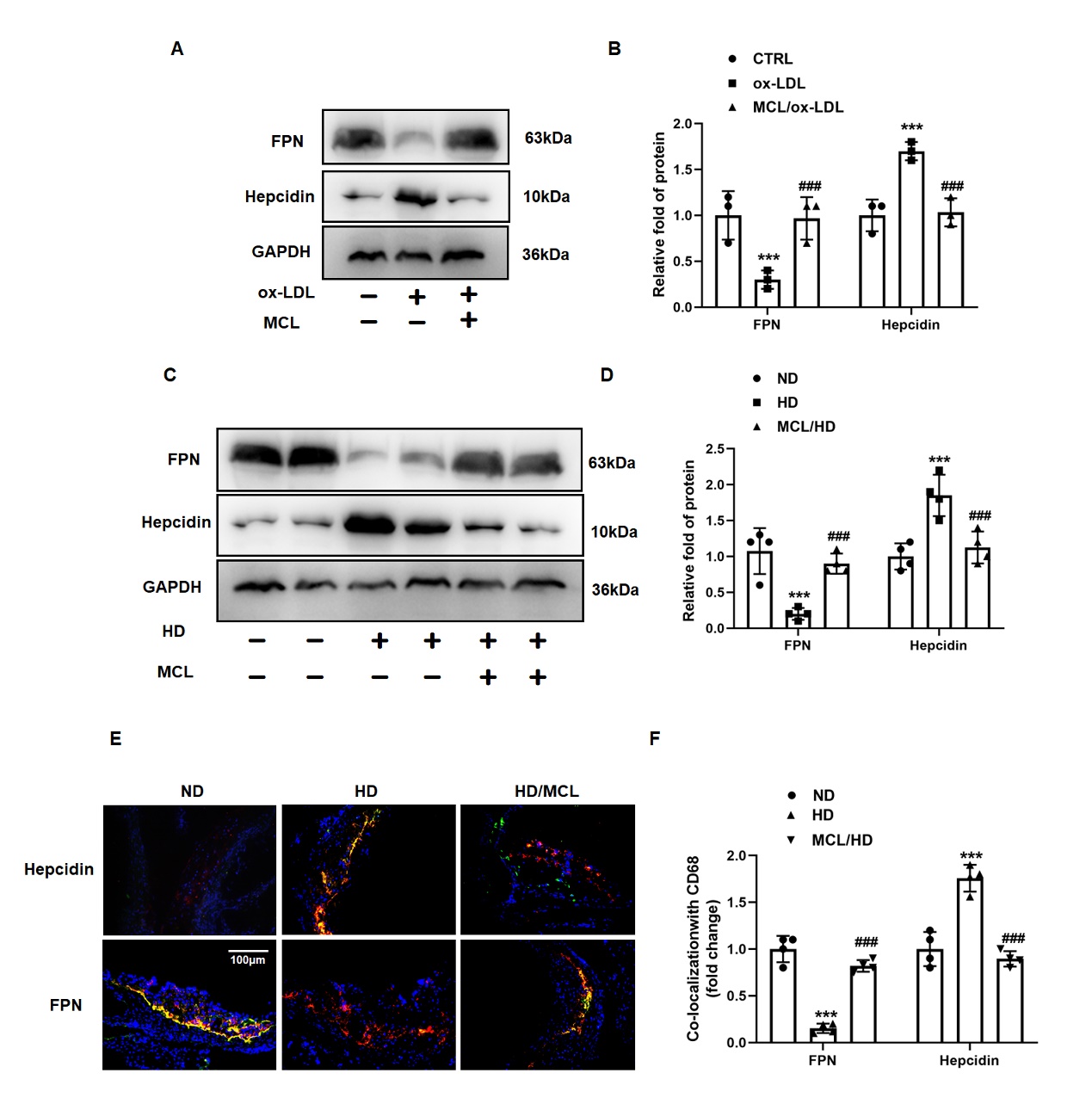


**Supplemental Fig. 4. MCL decreased hepcidin level and increased FPN level in macrophages**

(A, B) Macrophages were pretreated with ox-LDL (100 μg/ml) for 24 h, followed by MCL (10 μM) for 24 h. FPN and hepcidin level were detected. N=3. ***p < 0.001 vs CTRL group; ###p < 0.001 vs ox-LDL group. (C, D) FPN and hepcidin level in artery lysate were detected N=4. ***p < 0.001 vs CTRL group; ###p < 0.001 vs ox-LDL group. (E, F) Immunofluorescence was used to observe the levels of FPN and hepcidin in plaque macrophages. ***p < 0.001 vs CTRL group; ###p < 0.001 vs ox-LDL group.

**
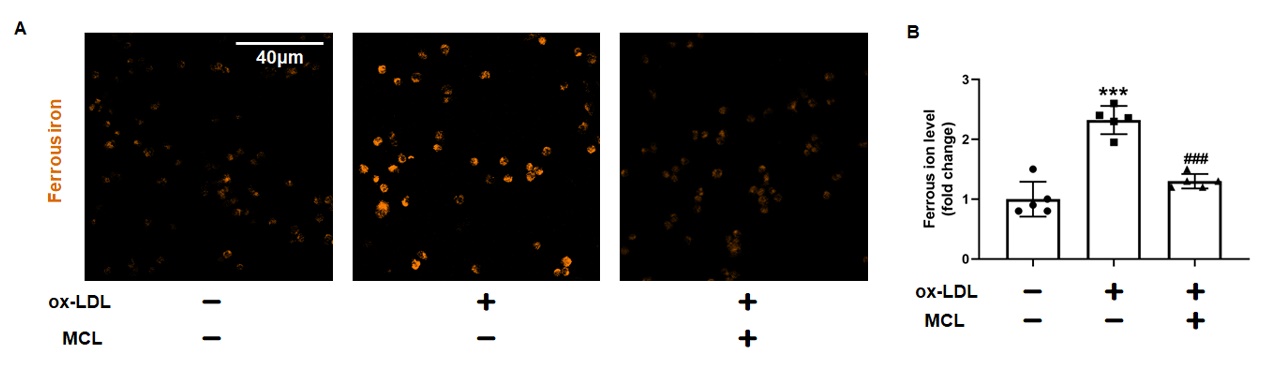
**

**Supplemental Fig. 5. Ferrous ion staining.**

THP-1 original macrophages are pretreated with MCL (10 μM) for 1 h, then stimulated with 100 μg/ml ox-LDL for 48 h. (A, B) Representative images and quantification of ferrous ion levels. N=5. ***p < 0.001 vs CTRL group; ###p < 0.001 vs ox-LDL group.

**
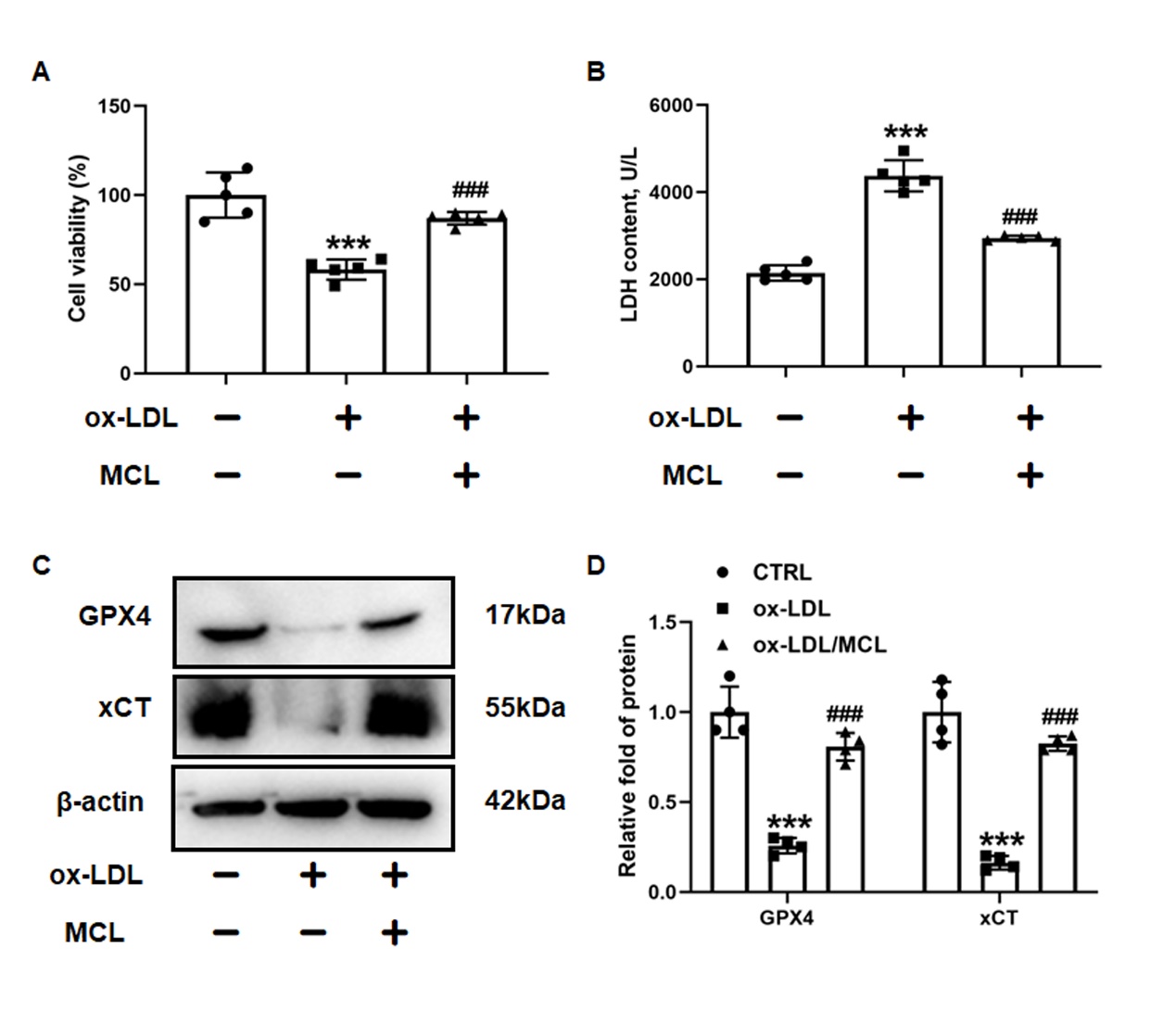
**

**Supplemental Fig 6. MCL reverses ox-LDL induced macrophage damage**

Macrophages were pretreated with ox-LDL (100 μg/ml) for 24 h, followed by MCL (10 μM) for 24 h. (A). CCK-8 assay was used to analysis cell viability. N=5; (B). LDH level in supernatant. N=5; (C-D) Total cell lysates were extracted, and protein levels of GPX4 and xCT were detected; N=4. ***p < 0.001 vs CTRL group; ###p < 0.001 vs ox-LDL group.

**
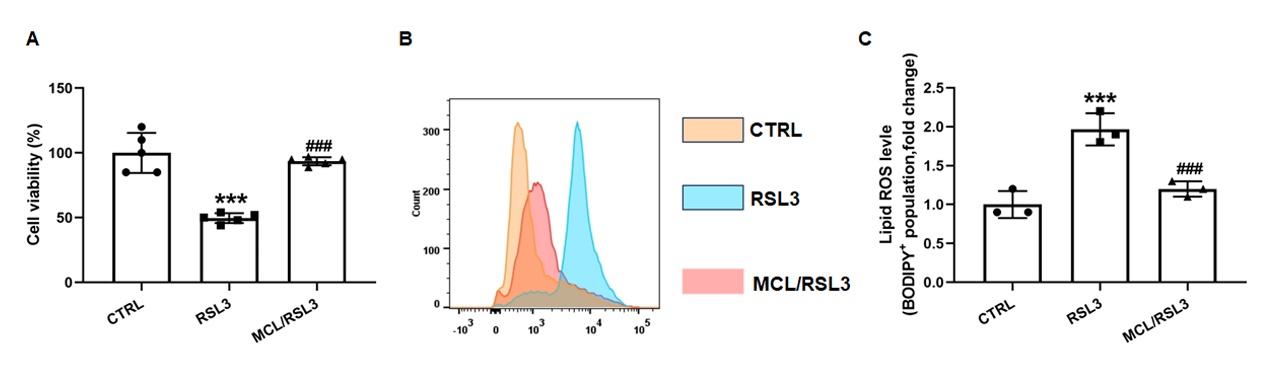
**

**Supplemental Fig 7. MCL inhibited macrophage ferroptosis induced by RSL3**

Macrophages were pretreated with MCL (10 μM) for 1 h, followed by RSL3 (5 μM) for 24 h. (A). CCK-8 assay was used to analysis cell viability. N=3; (B, C). The lipid ROS level was evaluated by flow cytometry using C11-Bodipy fluorescent probe. N=3; ***p < 0.001 vs CTRL group; ###p < 0.001 vs RSL3 group.

**
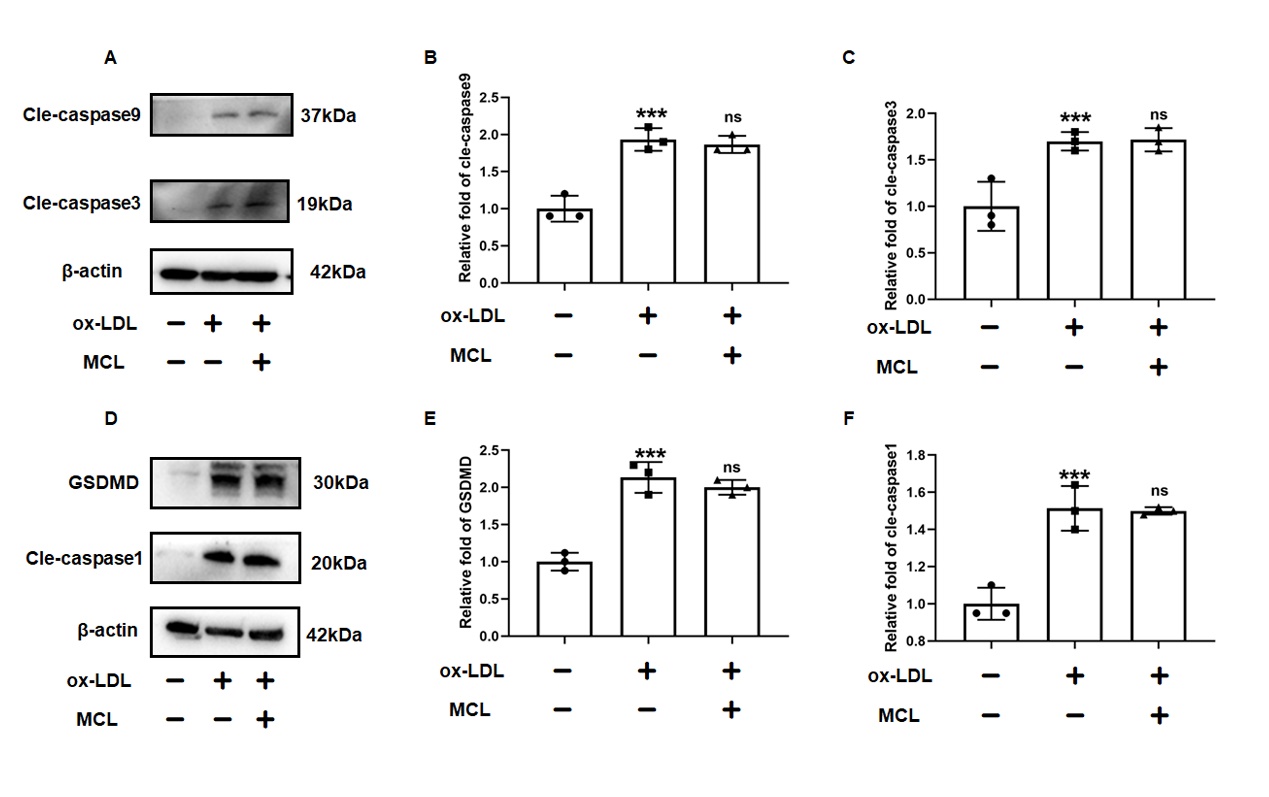
**

**Supplemental Fig 8. MCL cannot reduce cle-caspase3, cle-caspase9, cle-caspase1 and GSDMD level in macrophages induced by ox-LDL**

Macrophages were pretreated with MCL (10 μM) for 1 hour, then treated with ox-LDL (100 μg/ml) for 48 h. (A, B) Western blot is used to evaluate the level of cle-caspase3 and cle-caspase9.; (C, D) Western blot is used to evaluate the level of cle-caspase1 and GSDMD. ***p < 0.001 vs CTRL group;

**
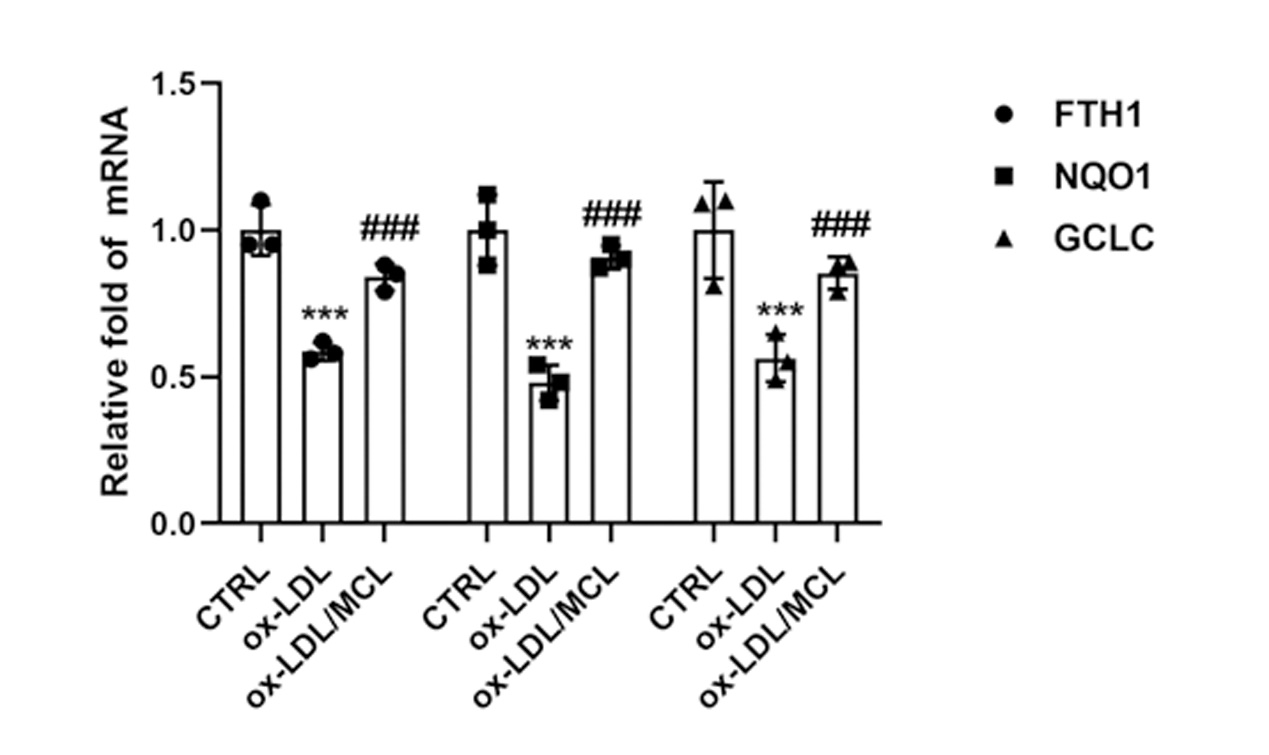
**

**Supplemental Fig 9. MCL increased the mRNA level of FTH1, GCLC and NQO1 in macrophages combined by ox-LDL.**

Macrophages were pretreated with MCL (10 μM) for 1 hour, then treated with ox-LDL (100 μg/ml) for 48 h. PCR was used to analysis mRNA level of FTH1, GCLC and NQO1. ***p < 0.001 vs CTRL group; ###p < 0.001 vs ox-LDL group.


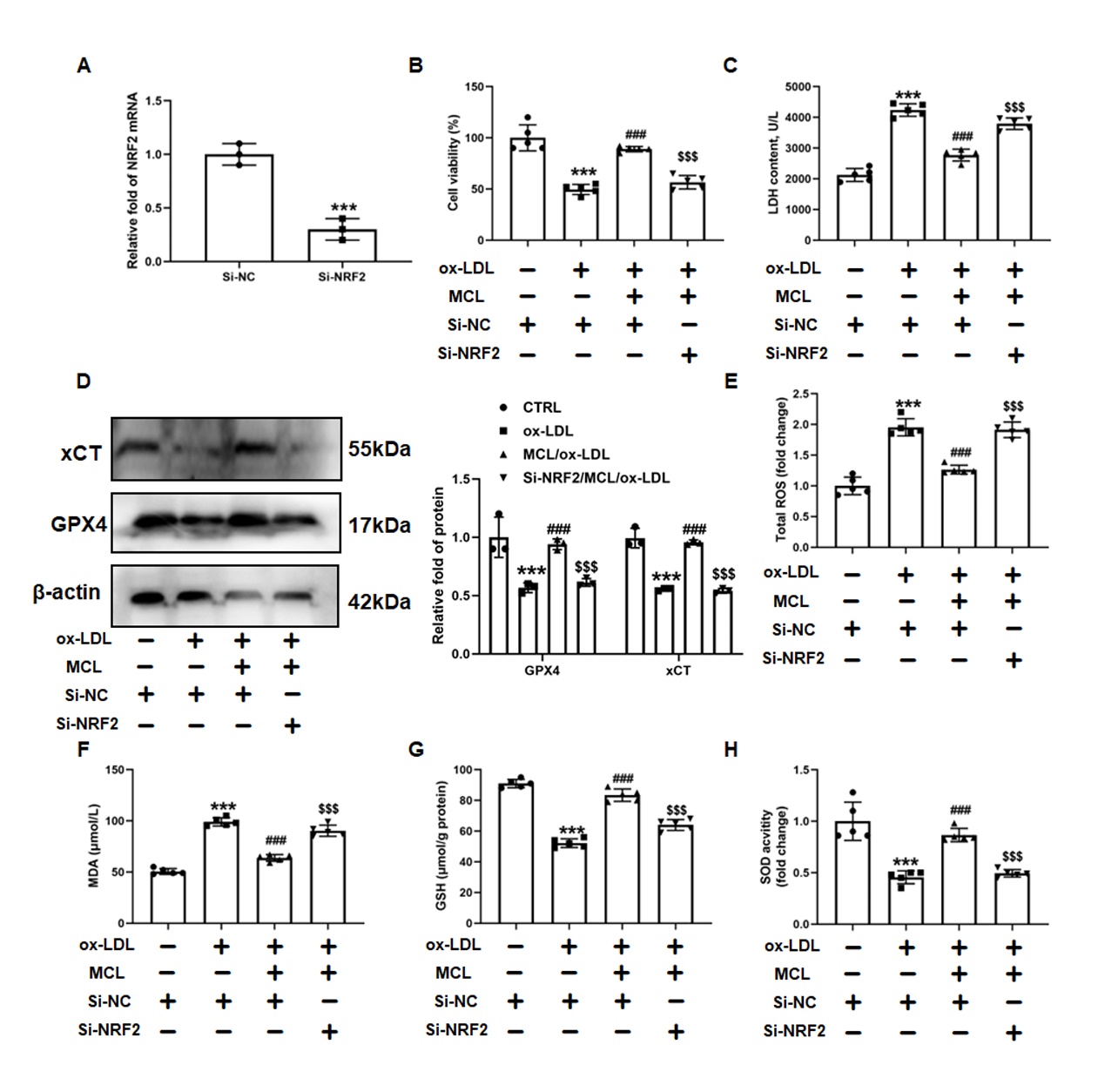


**Supplemental Fig.10. Si-NRF2 weaken the the effect of MCL on improving cell viability and increasing GPX4/xCT level.** Si-NRF2 was used to knock down NRF2 levels in macrophages. (A). PCR was used to evaluate NRF2 mRNA levels. N=3. ***, p<0.001.(B). CCK-8 assay was used to analysis cell viability. N=5; (C). LDH level in supernatant. N=5; (D) Total cell lysates were extracted, and protein levels of GPX4 and xCT were detected; N=3; (E) Total ROS level were detected. N=5; (F-H). The levels of MDA, GSH and SOD activity were measured in macrophages by commercial kits. ***p < 0.001 vs control group. ###p < 0.001 vs ox-LDL group. $$$p < 0.001 vs MCL + ox-LDL group.


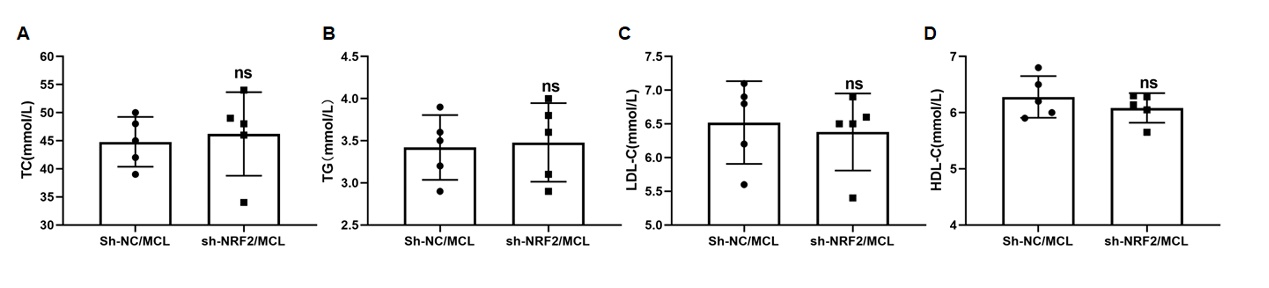


**Supplemental Fig.11. The serum lipid level in ApoE-/- with AAV-sh-NRF2 or AAV-sh-NC.** Blood lipid levels in the mice were measured. (A) TC level. N=5; (B) TG level. N=5; (C) LDL-C level. N=5; (D) HDL-C level. N=5;


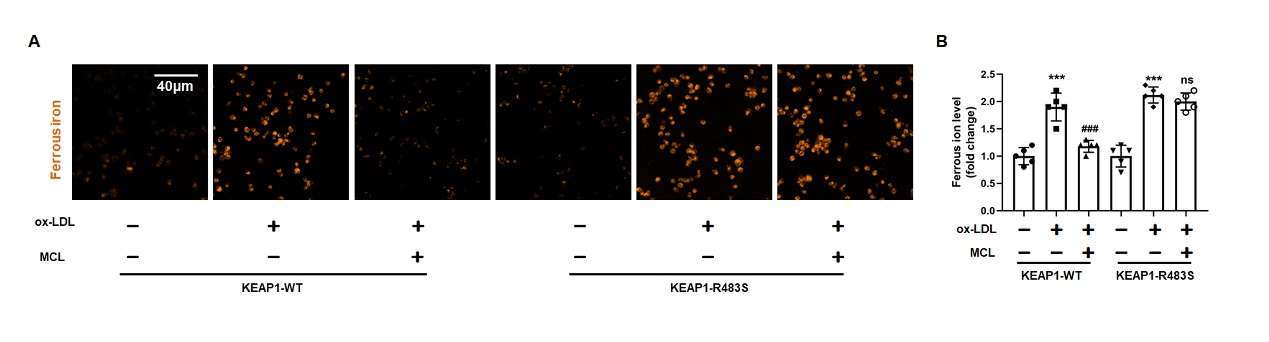


**Supplemental Fig. 12. Ferrous ion staining.**

Plasmid with mutated KEAP1 R483S are transfected into macrophages, followed by ox-LDL and MCL treated. (A, B) Representative images and quantification of ferrous ion levels. N=5. ***p < 0.001 vs CTRL group; ###p < 0.001 vs ox-LDL group.

**
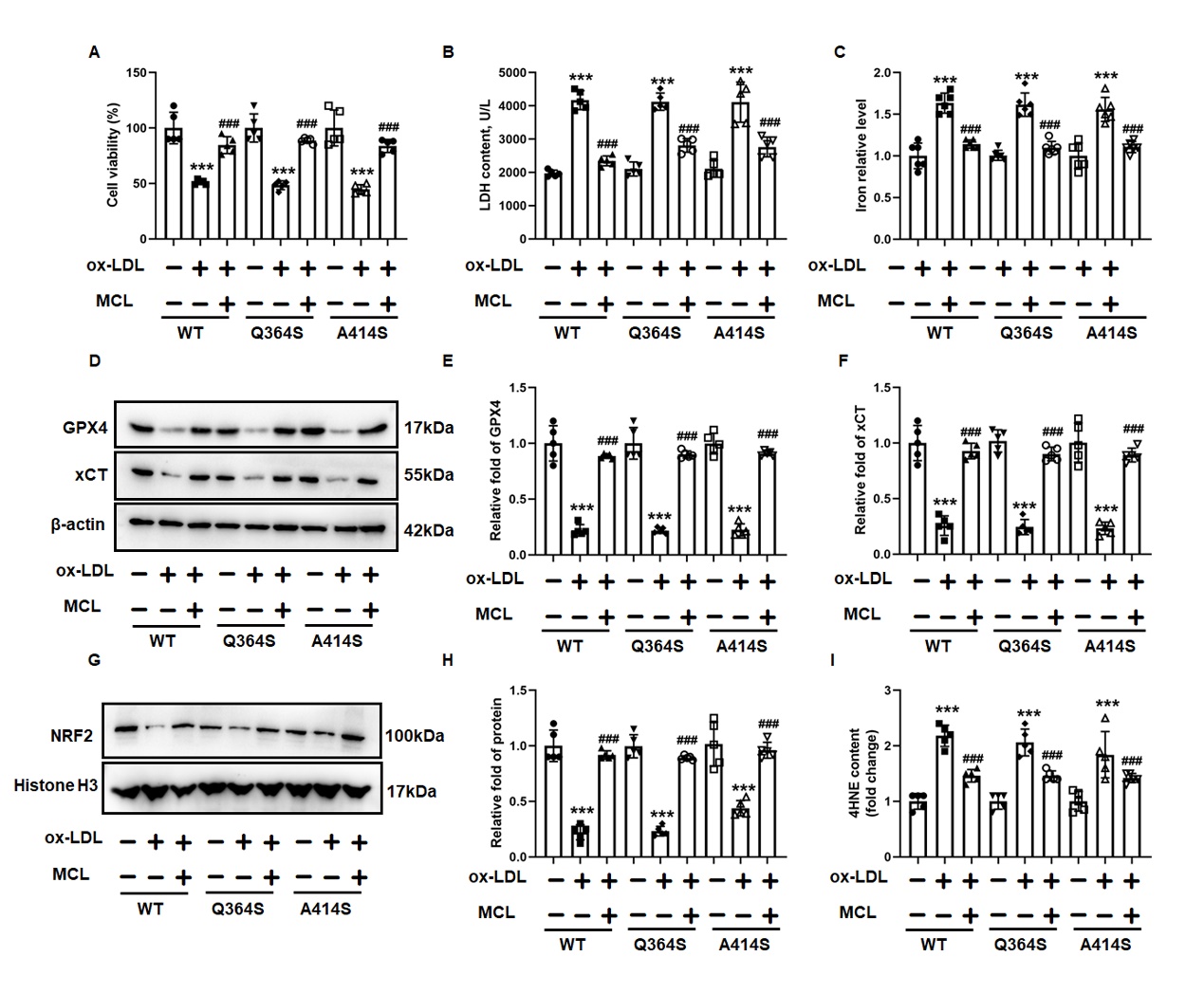
**

**Supplemental Fig 13. MCL inhibited macrophage ferroptosis and enhances NRF2 nuclear translocation in** **KEAP1-Q364S and KEAP1-A414S mutated macrophages.**

Plasmid with KEAP1-WT, mutated KEAP1-Q364S, and KEAP1-A414S are transfected into macrophages, followed by ox-LDL and MCL treated same as before. (A). CCK-8 assay was used to analysis cell viability. N=5; (B). LDH level in supernatant. N=5; (C) The levels of iron content were measured in macrophages by commercial kits. N=5; (D-F) Total cell lysates were extracted, and protein levels of GPX4 and xCT were detected; N=5. (G-I). The protein levels of NRF2 in macrophages. N=5; ***p < 0.001 vs CTRL group; ###p < 0.001 vs ox-LDL group.

**
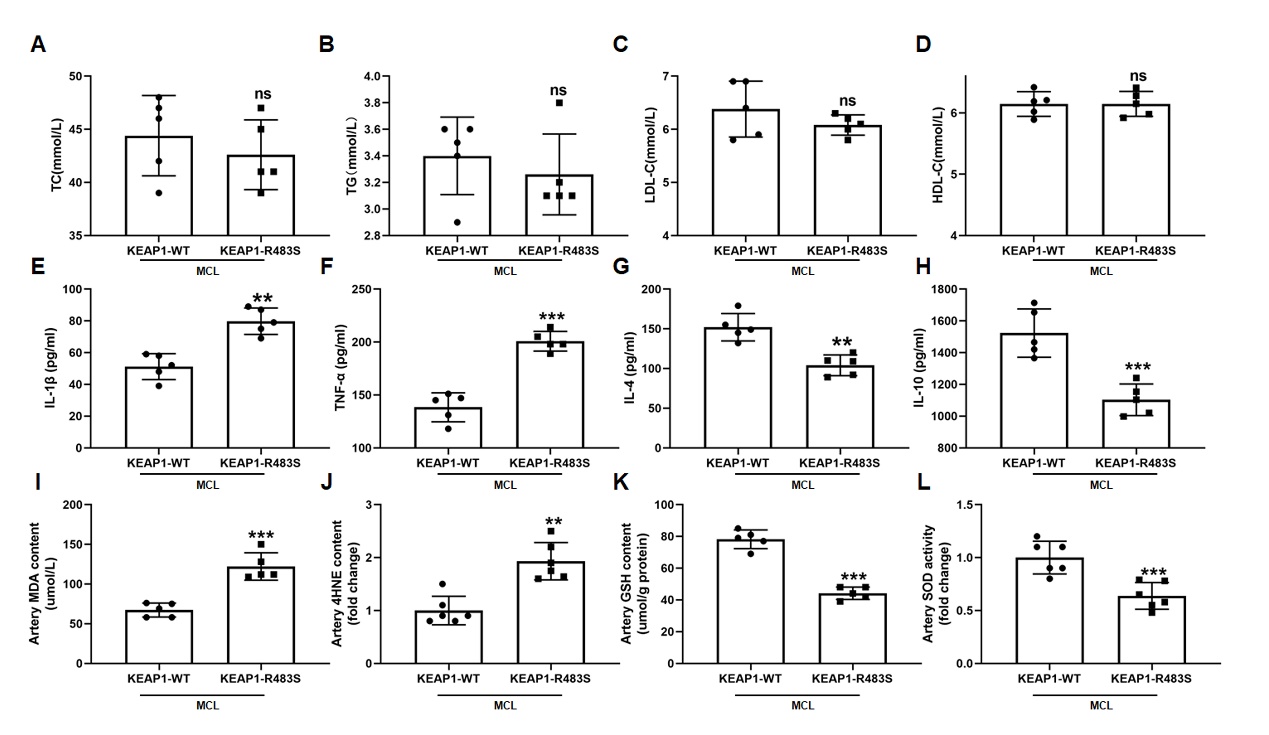
**

**Supplemental Fig. 14. Arg483 mutation weaken the anti-inflammatory and antioxidant effects of MCL, but does not affect the lipid-lowering effects.**

AAV-KEAP1-WT and AAV-KEAP1-R483S were injected intravenously through the tail of the mouse into 8-weeks-old ApoE-/- mice, all of which were received MCL gavage. (A) TC level. N=5; (B) TG level. N=5; (C) LDL-C level. N=5; (D) HDL-C level. N=5; (E). IL-1β level in serum. N=5; (F). TNF-α level in serum. N=5; (G). IL-4 level in serum. N=5; (H). IL-10 level in serum. N=5. (I) MDA levels in mouse arterial lysates. N=5; (J) 4-HNE levels in mouse arterial lysates. N=5; (K) GSH levels in mouse arterial lysates. N=5; (L) SOD activity in mouse arterial lysates. **p < 0.01, ***p < 0.001. vs KEAP1-WT group.


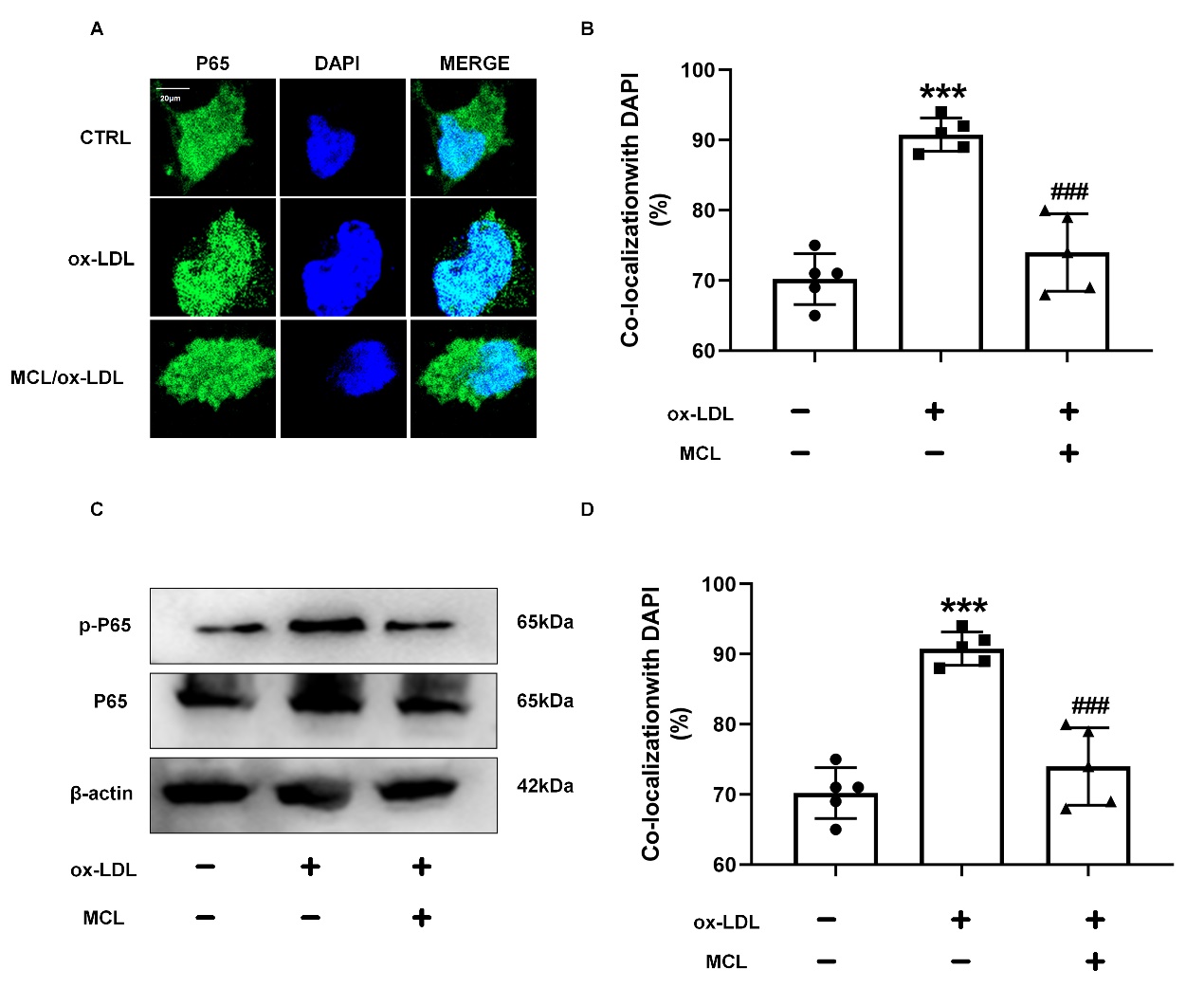


**Supplemental Fig 15. MCL reverses ox-LDL induced nuclear translocation and phosphorylation of P65**

Macrophages were pretreated with MCL (10 μM) for 1 hour, then treated with ox-LDL (100 μg/ml) for 48 h. (A, B) Confocal fluorescent staining is used to evaluate the nuclear translocation of NRF2. N=5; (C, D) Western blot is used to evaluate the level of p-P65. ***p < 0.001 vs CTRL group; ###p < 0.001 vs ox-LDL group.

**Supplemental Table 1: Protein and nucleic acid sequences of KEAP1-WT and KEAP1-R483S**

Keap1 kelch-like ECH-associated protein 1 [ Mus musculus (house mouse) ]

Gene ID: 50868 NM_016679.4 1875bp

**KEAP1-WT：**

ATGCAGCCCGAACCCAAGCTTAGCGGGGCTCCCCGCAGCAGCCAGTTCCTGCCCCTGTGGTCAAAGTGCCCCGAGGGGGCCGGGGACGCAGTGATGTATGCCTCCACGGAGTGCAAGGCAGAGGTGACGCCCTCGCAGGACGGTAACCGAACCTTCAGCTACACACTAGAGGATCACACCAAGCAGGCTTTTGGCGTCATGAACGAGCTTCGCCTGAGCCAGCAACTCTGTGACGTGACCCTGCAGGTCAAATATGAGGACATCCCAGCTGCCCAATTCATGGCTCACAAAGTGGTGCTGGCCTCCTCCAGCCCAGTCTTTAAAGCCATGTTCACCAACGGGCTTCGGGAGCAGGGCATGGAGGTGGTGTCCATCGAAGGCATCCACCCTAAGGTCATGGAAAGGCTTATTGAGTTCGCCTACACGGCCTCCATCTCCGTGGGCGAGAAGTGTGTCCTGCACGTGATGAACGGGGCGGTCATGTACCAGATTGACAGCGTGGTTCGAGCCTGCAGCGACTTCCTCGTGCAGCAGCTGGACCCCAGCAACGCCATTGGCATCGCCAACTTCGCGGAGCAGATCGGCTGCACTGAACTGCACCAGCGTGCCCGGGAGTATATCTACATGCACTTCGGGGAGGTGGCCAAGCAGGAGGAGTTCTTCAACCTGTCACACTGCCAGCTGGCCACGCTCATCAGCCGGGATGATCTGAACGTACGCTGCGAGTCCGAGGTGTTCCACGCGTGCATCGACTGGGTCAAATACGACTGCCCGCAGCGGCGCTTCTACGTGCAGGCACTGCTGCGGGCCGTGCGCTGCCATGCGCTCACGCCGCGCTTCCTGCAGACGCAGCTGCAGAAGTGTGAGATCCTGCAGGCCGACGCGCGCTGCAAGGACTACCTGGTGCAGATATTCCAGGAGCTCACGCTGCACAAGCCCACGCAGGCAGTGCCCTGCCGCGCGCCCAAAGTGGGCCGCCTCATCTACACAGCGGGCGGTTACTTCCGACAGTCGCTCAGCTACCTGGAGGCCTACAACCCGAGCAATGGCTCCTGGCTGCGCCTGGCCGATCTACAGGTGCCGCGCAGTGGGCTGGCAGGCTGCGTGGTGGGTGGGCTGCTATACGCTGTGGGCGGCCGCAACAACTCTCCGGATGGCAACACTGACTCCAGCGCCCTGGACTGCTACAACCCCATGACCAACCAGTGGTCGCCCTGTGCCTCTATGAGCGTGCCACGCAACCGCATCGGGGTGGGGGTCATAGATGGCCACATCTACGCAGTCGGGGGTTCCCACGGCTGCATCCACCACAGCAGCGTGGAGAGATATGAGCCAGAGCGGGACGAGTGGCATCTAGTCGCGCCAATGTTGACACGGAGGATTGGCGTGGGCGTGGCAGTGCTCAACCGCTTGCTGTATGCAGTGGGGGGCTTTGACGGGACTAACCGGCTTAACTCCGCAGAATGTTACTATCCAGAGAGGAATGAGTGGCGGATGATCACACCGATGAATACCATCCGGAGCGGGGCCGGGGTCTGCGTGCTGCACAACTGTATCTATGCAGCAGGGGGCTACGATGGGCAGGACCAGTTGAACAGTGTGGAGCGCTACGACGTGGAGACAGAGACCTGGACTTTCGTAGCCCCCATGAGGCATCACCGTAGTGCGCTGGGGATTACTGTGCACCAGGGCAAGATCTACGTCCTCGGAGGCTATGATGGCCACACTTTTCTGGACAGTGTGGAATGCTATGACCCGGACAGTGATACCTGGAGTGAGGTGACCCGCATGACATCTGGCCGCAGCGGGGTGGGTGTGGCCGTCACCATGGAACCCTGTCGGAAGCAAATTGATCAACAAAACTGTACCTGCTGA

**KEAP1-WT: Amino acid sequence**

MQPEPKLSGAPRSSQFLPLWSKCPEGAGDAVMYASTECKAEVTPSQDGNRTFSYTLEDHTKQAFGVMNELRLSQQLCDVTLQVKYEDIPAAQFMAHKVVLASSSPVFKAMFTNGLREQGMEVVSIEGIHPKVMERLIEFAYTASISVGEKCVLHVMNGAVMYQIDSVVRACSDFLVQQLDPSNAIGIANFAEQIGCTELHQRAREYIYMHFGEVAKQEEFFNLSHCQLATLISRDDLNVRCESEVFHACIDWVKYDCPQRRFYVQALLRAVRCHALTPRFLQTQLQKCEILQADARCKDYLVQIFQELTLHKPTQAVPCRAPKVGRLIYTAGGYFRQSLSYLEAYNPSNGSWLRLADLQVPRSGLAGCVVGGLLYAVGGRNNSPDGNTDSSALDCYNPMTNQWSPCASMSVPRNRIGVGVIDGHIYAVGGSHGCIHHSSVERYEPERDEWHLVAPMLTRRIGVGVAVLNRLLYAVGGFDGTNRLNSAECYYPERNEWRMITPMNTIRSGAGVCVLHNCIYAAGGYDGQDQLNSVERYDVETETWTFVAPMRHHRSALGITVHQGKIYVLGGYDGHTFLDSVECYDPDSDTWSEVTRMTSGRSGVGVAVTMEPCRKQIDQQNCTC

**KEAP1-R483S:** ATGCAGCCCGAACCCAAGCTTAGCGGGGCTCCCCGCAGCAGCCAGTTCCTGCCCCTGTGGTCAAAGTGCCCCGAGGGGGCCGGGGACGCAGTGATGTATGCCTCCACGGAGTGCAAGGCAGAGGTGACGCCCTCGCAGGACGGTAACCGAACCTTCAGCTACACACTAGAGGATCACACCAAGCAGGCTTTTGGCGTCATGAACGAGCTTCGCCTGAGCCAGCAACTCTGTGACGTGACCCTGCAGGTCAAATATGAGGACATCCCAGCTGCCCAATTCATGGCTCACAAAGTGGTGCTGGCCTCCTCCAGCCCAGTCTTTAAAGCCATGTTCACCAACGGGCTTCGGGAGCAGGGCATGGAGGTGGTGTCCATCGAAGGCATCCACCCTAAGGTCATGGAAAGGCTTATTGAGTTCGCCTACACGGCCTCCATCTCCGTGGGCGAGAAGTGTGTCCTGCACGTGATGAACGGGGCGGTCATGTACCAGATTGACAGCGTGGTTCGAGCCTGCAGCGACTTCCTCGTGCAGCAGCTGGACCCCAGCAACGCCATTGGCATCGCCAACTTCGCGGAGCAGATCGGCTGCACTGAACTGCACCAGCGTGCCCGGGAGTATATCTACATGCACTTCGGGGAGGTGGCCAAGCAGGAGGAGTTCTTCAACCTGTCACACTGCCAGCTGGCCACGCTCATCAGCCGGGATGATCTGAACGTACGCTGCGAGTCCGAGGTGTTCCACGCGTGCATCGACTGGGTCAAATACGACTGCCCGCAGCGGCGCTTCTACGTGCAGGCACTGCTGCGGGCCGTGCGCTGCCATGCGCTCACGCCGCGCTTCCTGCAGACGCAGCTGCAGAAGTGTGAGATCCTGCAGGCCGACGCGCGCTGCAAGGACTACCTGGTGCAGATATTCCAGGAGCTCACGCTGCACAAGCCCACGCAGGCAGTGCCCTGCCGCGCGCCCAAAGTGGGCCGCCTCATCTACACAGCGGGCGGTTACTTCCGACAGTCGCTCAGCTACCTGGAGGCCTACAACCCGAGCAATGGCTCCTGGCTGCGCCTGGCCGATCTACAGGTGCCGCGCAGTGGGCTGGCAGGCTGCGTGGTGGGTGGGCTGCTATACGCTGTGGGCGGCCGCAACAACTCTCCGGATGGCAACACTGACTCCAGCGCCCTGGACTGCTACAACCCCATGACCAACCAGTGGTCGCCCTGTGCCTCTATGAGCGTGCCACGCAACCGCATCGGGGTGGGGGTCATAGATGGCCACATCTACGCAGTCGGGGGTTCCCACGGCTGCATCCACCACAGCAGCGTGGAGAGATATGAGCCAGAGCGGGACGAGTGGCATCTAGTCGCGCCAATGTTGACACGGAGGATTGGCGTGGGCGTGGCAGTGCTCAACCGCTTGCTGTATGCAGTGGGGGGCTTTGACGGGACTAACTCGCTTAACTCCGCAGAATGTTACTATCCAGAGAGGAATGAGTGGCGGATGATCACACCGATGAATACCATCCGGAGCGGGGCCGGGGTCTGCGTGCTGCACAACTGTATCTATGCAGCAGGGGGCTACGATGGGCAGGACCAGTTGAACAGTGTGGAGCGCTACGACGTGGAGACAGAGACCTGGACTTTCGTAGCCCCCATGAGGCATCACCGTAGTGCGCTGGGGATTACTGTGCACCAGGGCAAGATCTACGTCCTCGGAGGCTATGATGGCCACACTTTTCTGGACAGTGTGGAATGCTATGACCCGGACAGTGATACCTGGAGTGAGGTGACCCGCATGACATCTGGCCGCAGCGGGGTGGGTGTGGCCGTCACCATGGAACCCTGTCGGAAGCAAATTGATCAACAAAACTGTACCTGCTGA

**KEAP1-R483S: Amino acid sequence**

MQPEPKLSGAPRSSQFLPLWSKCPEGAGDAVMYASTECKAEVTPSQDGNRTFSYTLEDHTKQAFGVMNELRLSQQLCDVTLQVKYEDIPAAQFMAHKVVLASSSPVFKAMFTNGLREQGMEVVSIEGIHPKVMERLIEFAYTASISVGEKCVLHVMNGAVMYQIDSVVRACSDFLVQQLDPSNAIGIANFAEQIGCTELHQRAREYIYMHFGEVAKQEEFFNLSHCQLATLISRDDLNVRCESEVFHACIDWVKYDCPQRRFYVQALLRAVRCHALTPRFLQTQLQKCEILQADARCKDYLVQIFQELTLHKPTQAVPCRAPKVGRLIYTAGGYFRQSLSYLEAYNPSNGSWLRLADLQVPRSGLAGCVVGGLLYAVGGRNNSPDGNTDSSALDCYNPMTNQWSPCASMSVPRNRIGVGVIDGHIYAVGGSHGCIHHSSVERYEPERDEWHLVAPMLTRRIGVGVAVLNRLLYAVGGFDGTNSLNSAECYYPERNEWRMITPMNTIRSGAGVCVLHNCIYAAGGYDGQDQLNSVERYDVETETWTFVAPMRHHRSALGITVHQGKIYVLGGYDGHTFLDSVECYDPDSDTWSEVTRMTSGRSGVGVAVTMEPCRKQIDQQNCTC

**KEAP1 R483S**

**KEAP1 kelch like ECH associated protein 1 [ *Homo sapiens* (human)]**

Gene ID: 9817 NM_012289.4 CDS=1875bp

ATGCAGCCAGATCCCAGGCCTAGCGGGGCTGGGGCCTGCTGCCGATTCCTGCCCCTGCAGTCACAGTGCCCTGAGGGGGCAGGGGACGCGGTGATGTACGCCTCCACTGAGTGCAAGGCGGAGGTGACGCCCTCCCAGCATGGCAACCGCACCTTCAGCTACACCCTGGAGGATCATACCAAGCAGGCCTTTGGCATCATGAACGAGCTGCGGCTCAGCCAGCAGCTGTGTGACGTCACACTGCAGGTCAAGTACCAGGATGCACCGGCCGCCCAGTTCATGGCCCACAAGGTGGTGCTGGCCTCATCCAGCCCTGTCTTCAAGGCCATGTTCACCAACGGGCTGCGGGAGCAGGGCATGGAGGTGGTGTCCATTGAGGGTATCCACCCCAAGGTCATGGAGCGCCTCATTGAATTCGCCTACACGGCCTCCATCTCCATGGGCGAGAAGTGTGTCCTCCACGTCATGAACGGTGCTGTCATGTACCAGATCGACAGCGTTGTCCGTGCCTGCAGTGACTTCCTGGTGCAGCAGCTGGACCCCAGCAATGCCATCGGCATCGCCAACTTCGCTGAGCAGATTGGCTGTGTGGAGTTGCACCAGCGTGCCCGGGAGTACATCTACATGCATTTTGGGGAGGTGGCCAAGCAAGAGGAGTTCTTCAACCTGTCCCACTGCCAACTGGTGACCCTCATCAGCCGGGACGACCTGAACGTGCGCTGCGAGTCCGAGGTCTTCCACGCCTGCATCAACTGGGTCAAGTACGACTGCGAACAGCGACGGTTCTACGTCCAGGCGCTGCTGCGGGCCGTGCGCTGCCACTCGTTGACGCCGAACTTCCTGCAGATGCAGCTGCAGAAGTGCGAGATCCTGCAGTCCGACTCCCGCTGCAAGGACTACCTGGTCAAGATCTTCGAGGAGCTCACCCTGCACAAGCCCACGCAGGTGATGCCCTGCCGGGCGCCCAAGGTGGGCCGCCTGATCTACACCGCGGGCGGCTACTTCCGACAGTCGCTCAGCTACCTGGAGGCTTACAACCCCAGTGACGGCACCTGGCTCCGGTTGGCGGACCTGCAGGTGCCGCGGAGCGGCCTGGCCGGCTGCGTGGTGGGCGGGCTGTTGTACGCCGTGGGCGGCAGGAACAACTCGCCCGACGGCAACACCGACTCCAGCGCCCTGGACTGTTACAACCCCATGACCAATCAGTGGTCGCCCTGCGCCCCCATGAGCGTGCCCCGTAACCGCATCGGGGTGGGGGTCATCGATGGCCACATCTATGCCGTCGGCGGCTCCCACGGCTGCATCCACCACAACAGTGTGGAGAGGTATGAGCCAGAGCGGGATGAGTGGCACTTGGTGGCCCCAATGCTGACACGAAGGATCGGGGTGGGCGTGGCTGTCCTCAATCGTCTCCTTTATGCCGTGGGGGGCTTTGACGGGACAAACAGCCTTAATTCAGCTGAGTGTTACTACCCAGAGAGGAACGAGTGGCGAATGATCACAGCAATGAACACCATCCGAAGCGGGGCAGGCGTCTGCGTCCTGCACAACTGTATCTATGCTGCTGGGGGCTATGATGGTCAGGACCAGCTGAACAGCGTGGAGCGCTACGATGTGGAAACAGAGACGTGGACTTTCGTAGCCCCCATGAAGCACCGGCGAAGTGCCCTGGGGATCACTGTCCACCAGGGGAGAATCTACGTCCTTGGAGGCTATGATGGTCACACGTTCCTGGACAGTGTGGAGTGTTACGACCCAGATACAGACACCTGGAGCGAGGTGACCCGAATGACATCGGGCCGGAGTGGGGTGGGCGTGGCTGTCACCATGGAGCCCTGCCGGAAGCAGATTGACCAGCAGAACTGTACCTGTTGA

Amino acid sequence：

MQPDPRPSGAGACCRFLPLQSQCPEGAGDAVMYASTECKAEVTPSQHGNRTFSYTLEDHTKQAFGIMNELRLSQQLCDVTLQVKYQDAPAAQFMAHKVVLASSSPVFKAMFTNGLREQGMEVVSIEGIHPKVMERLIEFAYTASISMGEKCVLHVMNGAVMYQIDSVVRACSDFLVQQLDPSNAIGIANFAEQIGCVELHQRAREYIYMHFGEVAKQEEFFNLSHCQLVTLISRDDLNVRCESEVFHACINWVKYDCEQRRFYVQALLRAVRCHSLTPNFLQMQLQKCEILQSDSRCKDYLVKIFEELTLHKPTQVMPCRAPKVGRLIYTAGGYFRQSLSYLEAYNPSDGTWLRLADLQVPRSGLAGCVVGGLLYAVGGRNNSPDGNTDSSALDCYNPMTNQWSPCAPMSVPRNRIGVGVIDGHIYAVGGSHGCIHHNSVERYEPERDEWHLVAPMLTRRIGVGVAVLNRLLYAVGGFDGTNSLNSAECYYPERNEWRMITAMNTIRSGAGVCVLHNCIYAAGGYDGQDQLNSVERYDVETETWTFVAPMKHRRSALGITVHQGRIYVLGGYDGHTFLDSVECYDPDTDTWSEVTRMTSGRSGVGVAVTMEPCRKQIDQQNCTC

**Supplemental Table 2 The primer sequence**

| Primer | Sequence（5' to 3'） |
| --- | --- |
| GPX4-hum-F | GAGGCAAGACCGAAGTAAACTAC |
| GPX4-hum-R | CCGAACTGGTTACACGGGAA |
| GPX4-mus-F | TGTGCATCCCGCGATGATT |
| GPX4-mus-R | CCCTGTACTTATCCAGGCAGA |
| xCT-hum-F | TCTCCAAAGGAGGTTACCTGC |
| xCT-hum-R | AGACTCCCCTCAGTAAAGTGAC |
| xCT-mus-F | GGCACCGTCATCGGATCAG |
| xCT-mus-R | CTCCACAGGCAGACCAGAAAA |
| GAPDH-hum-F | GGAGCGAGATCCCTCCAAAAT |
| GAPDH-hum-R | GGCTGTTGTCATACTTCTCATGG |
| GAPDH-mus-F | AGGTCGGTGTGAACGGATTTG |
| GAPDH-mus-R  GCLC-hum-F  GCLC-hum-R  FTH1-hum-F  FTH1-hum-R  NQO1- hum-F  NQO1- hum-R | GGGGTCGTTGATGGCAACA  TGTCCGAGTTCAATACAGTTGAGCLC  ACAGCCTAATCTGGGAAATGAA  CTCCTACGTTTACCTGTCCATG  CAAGTCATCAGGCACATACAAG  AGCCGCAGACCTTGTGATATTCC  ATGGCAGCGTAAGTGTAAGCAAAC |
