# Supplementary material for: MCL attenuates atherosclerosis by suppressing macrophage ferroptosis via targeting KEAP1/NRF2 interaction
Source: Redox Biol. 2023 Dec 7;69:102987. doi: 10.1016/j.redox.2023.102987 (PMC10761782; doi:10.1016/j.redox.2023.102987)
Supplement: Multimedia component 2 [file mmc2.pdf]

Figure 2C

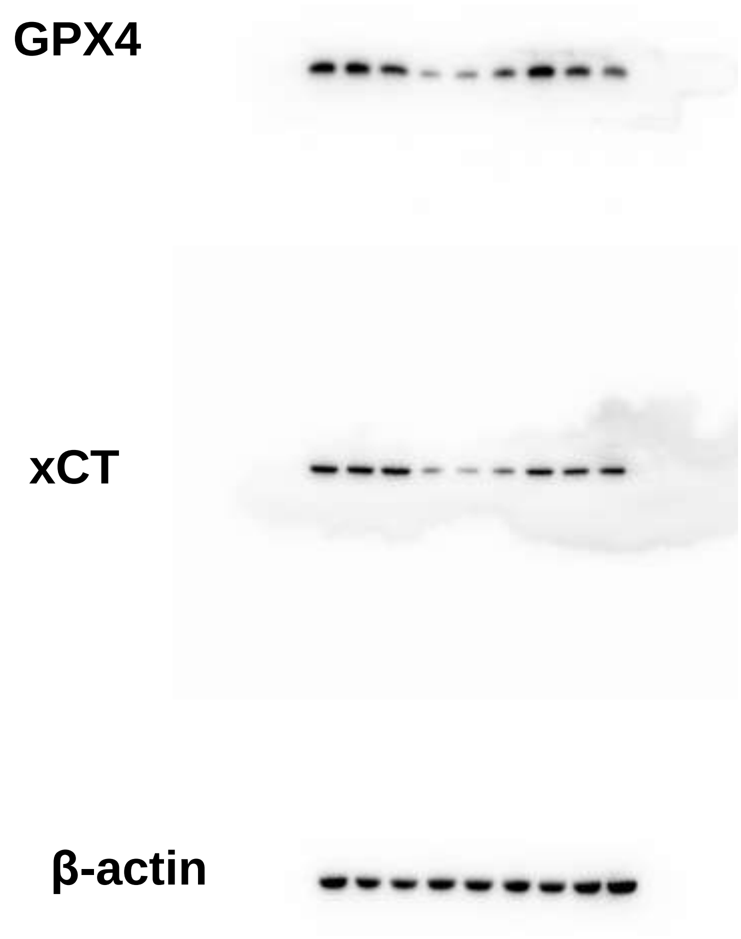

Figure 3I

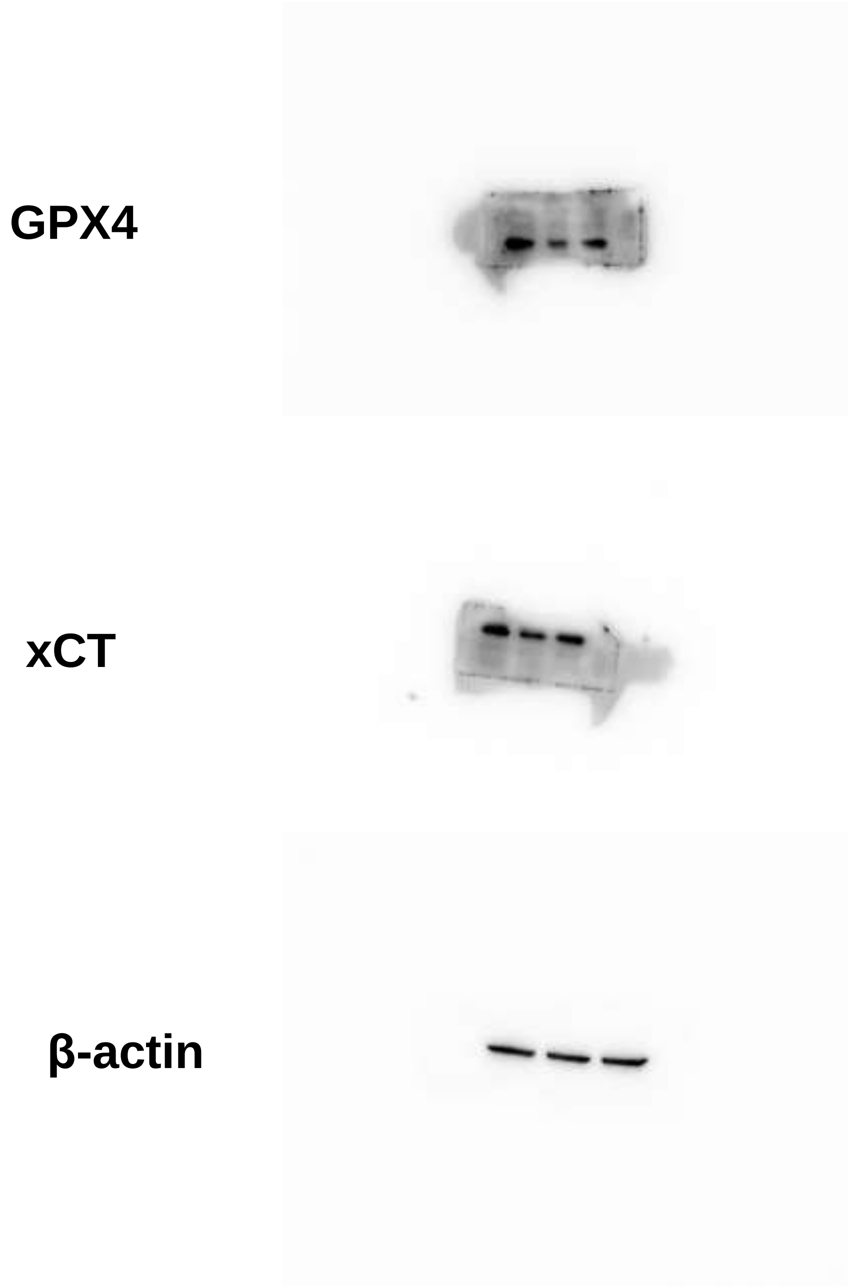

Figure 5A

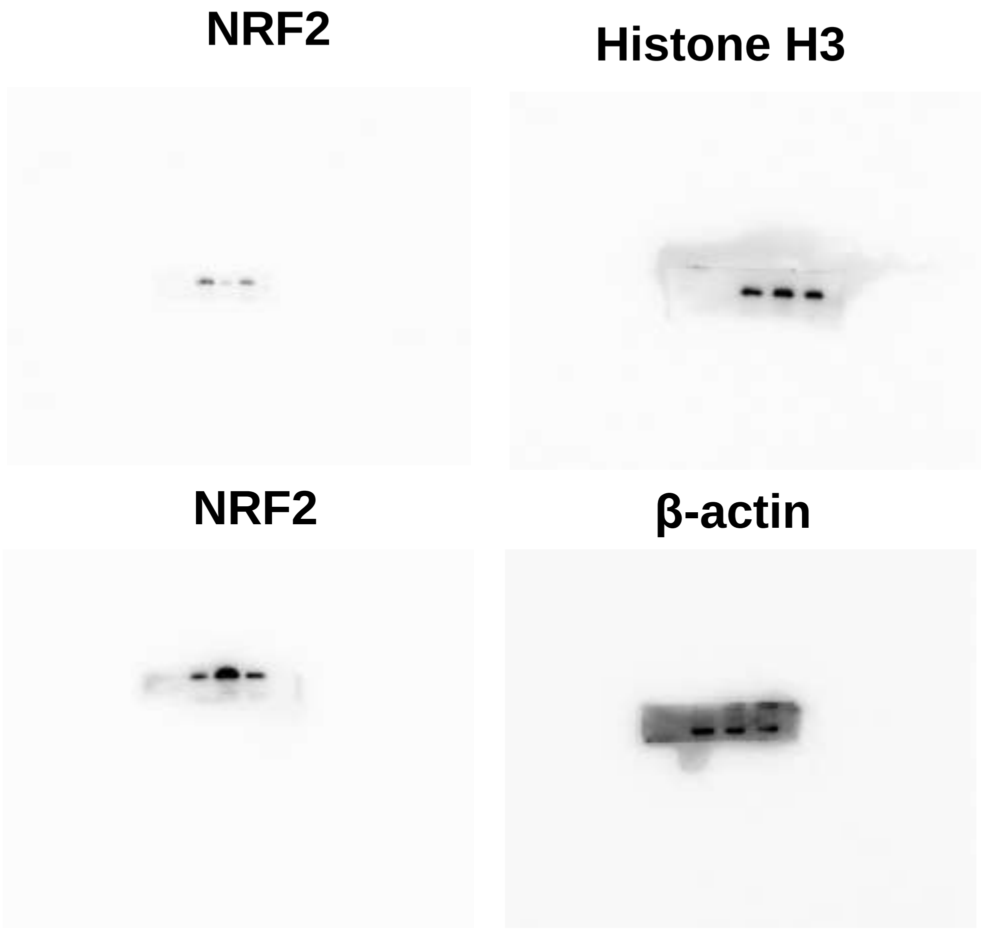

Figure 5E

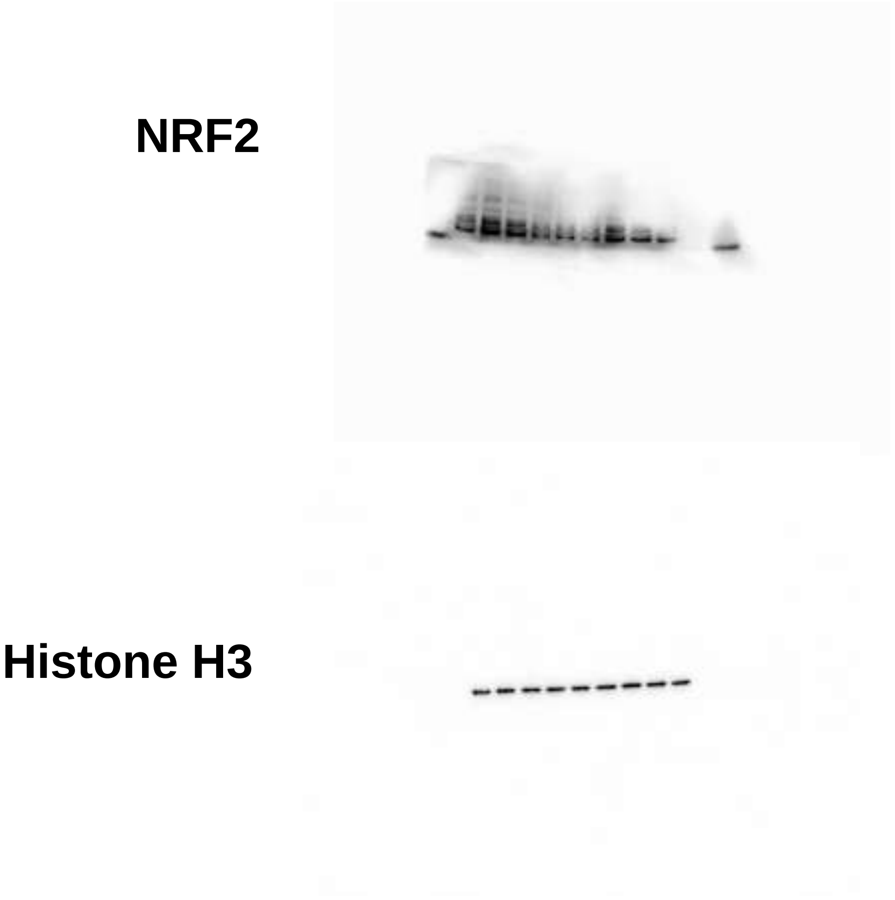

Figure 6G

GPX4

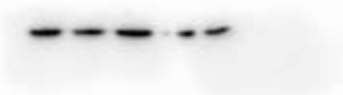

xCT

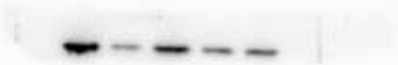

β-actin

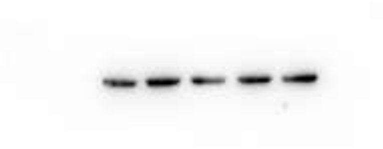

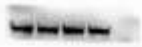

Input: NRF2

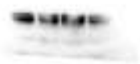

KEAP1

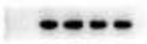

β-actin

Figure 8A

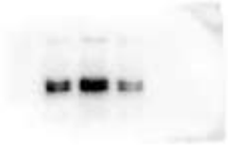

IB: KEAP1

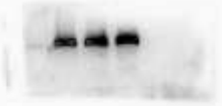

IP: NRF2

Figure 8F

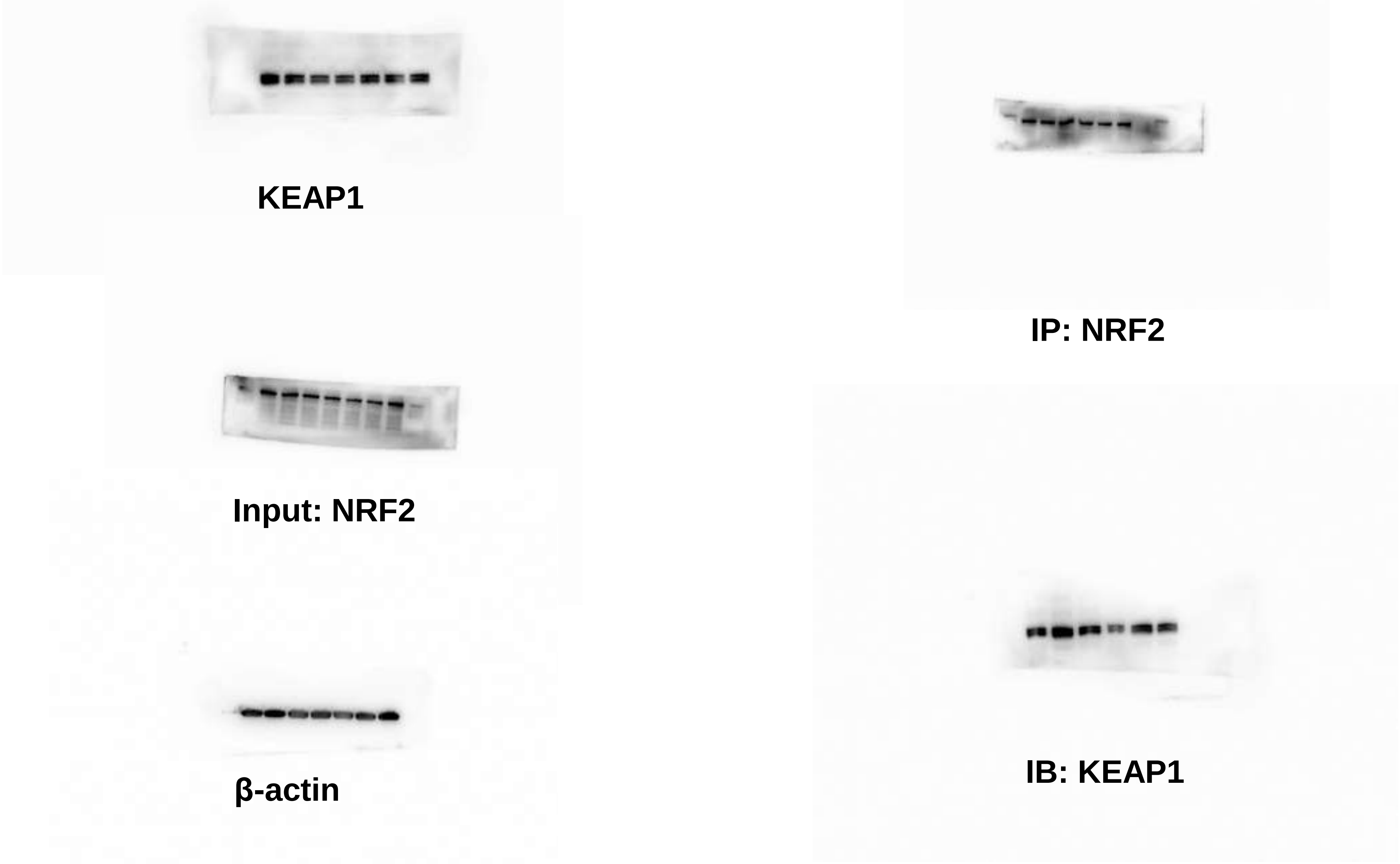

Figure 8H

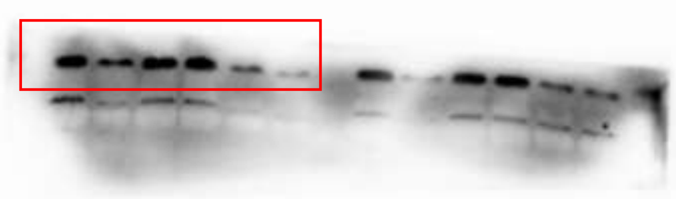

NRF2

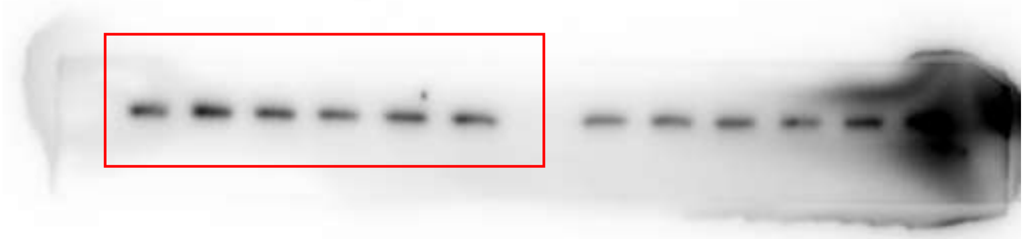

Histone H3

Figure 9E

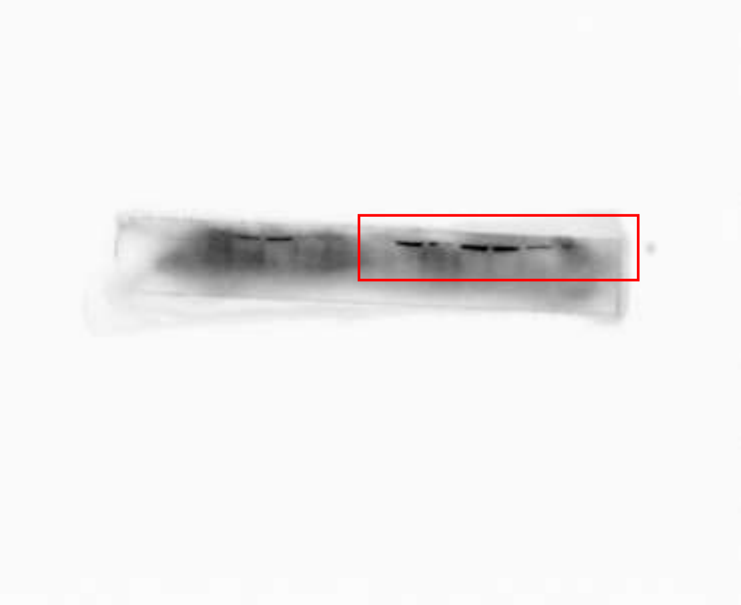

xCT

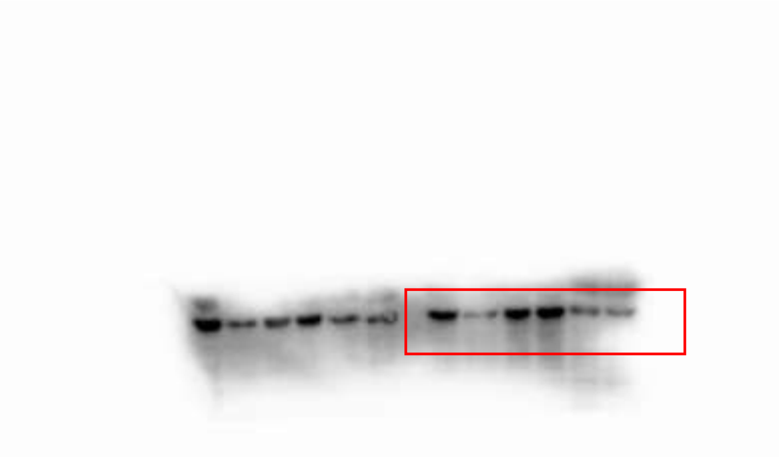

GPX4

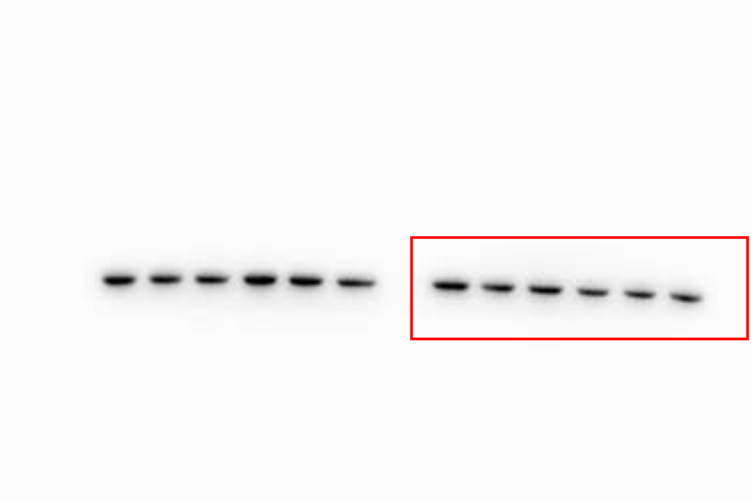

β-actin

Figure 11H

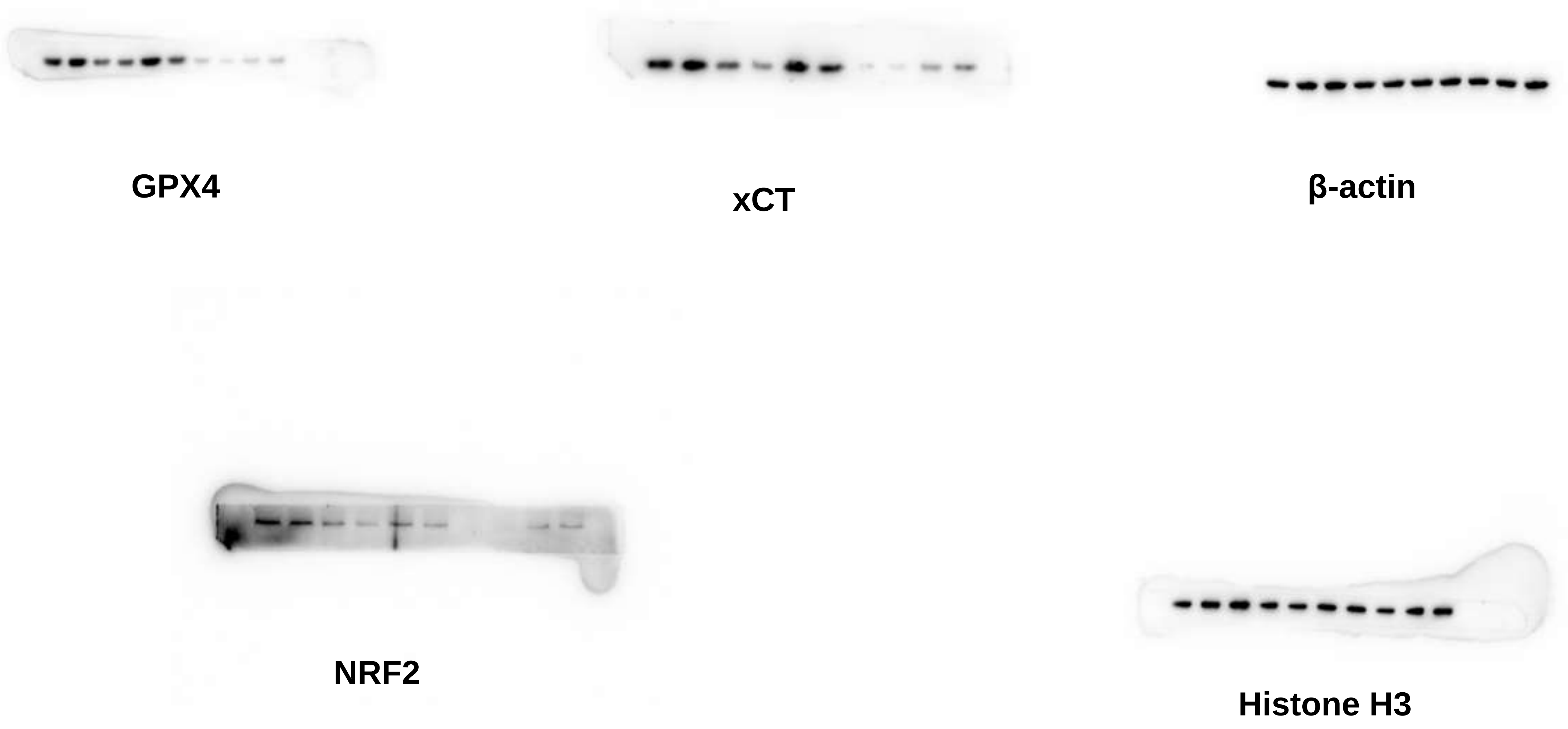

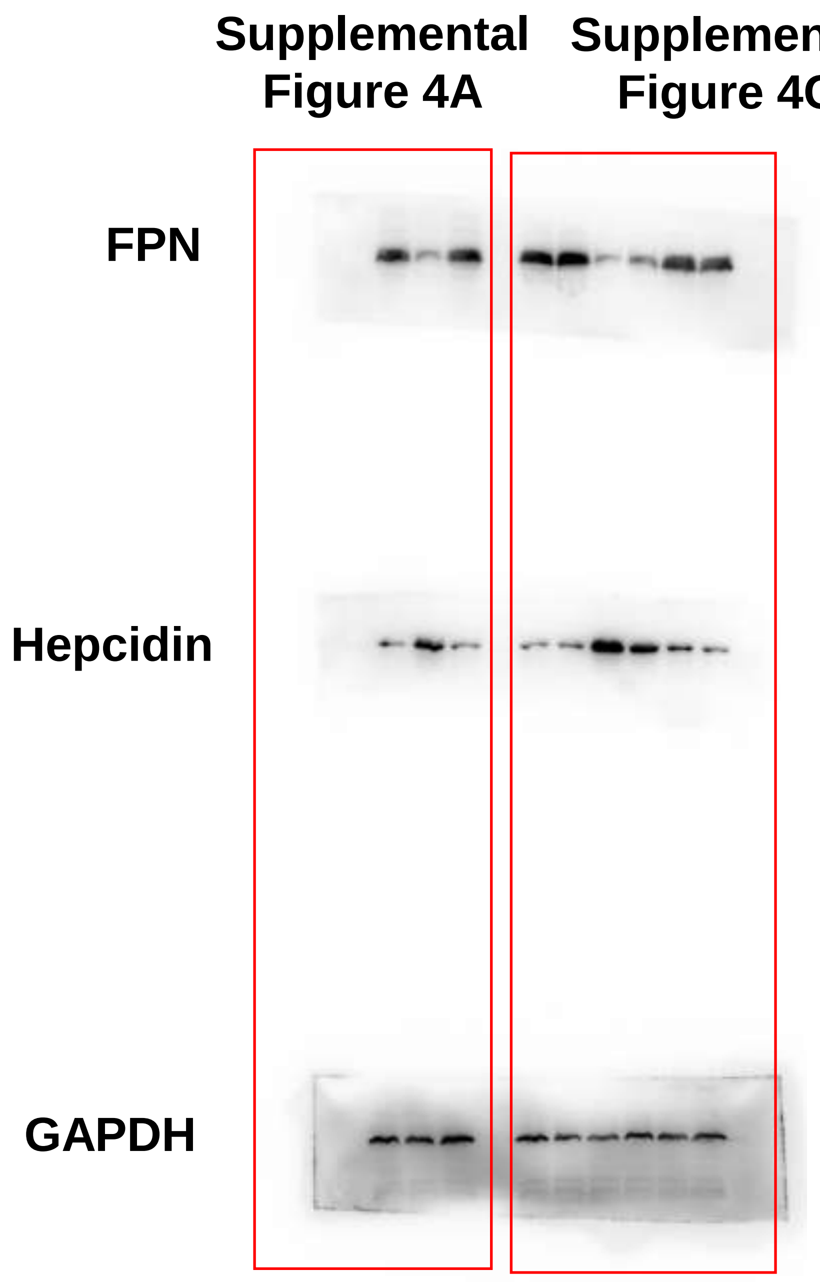
Supplemental Figure 4A
Supplemental Figure 4C
FPN
Hepcidin
GAPDH

Supplemental Figure 6C

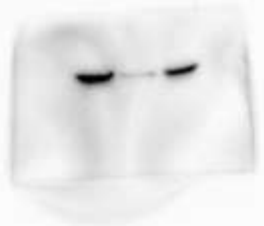

GPX4

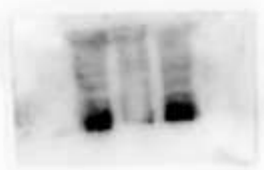

xCT

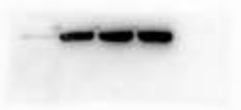

β-actin

Supplemental Figure 8A

Cle-caspase3

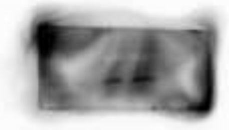

β-actin

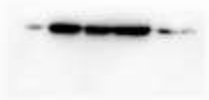

Cle-caspase9

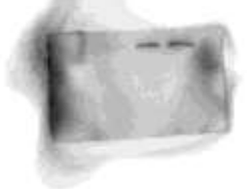

Supplemental Figure 8D

GSDMD

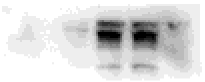

β-actin

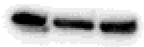

Cle-caspase1

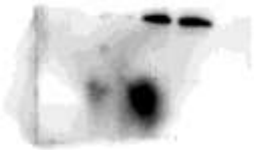

Supplemental Figure 10D

GPX4

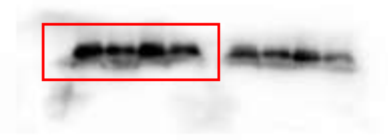

xCT

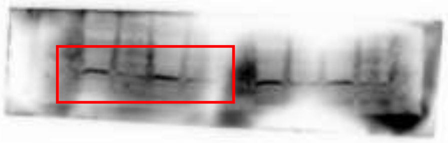

β-actin

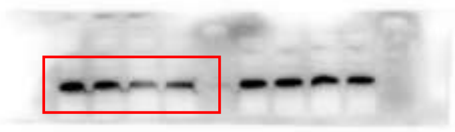

Supplemental Figure 13D

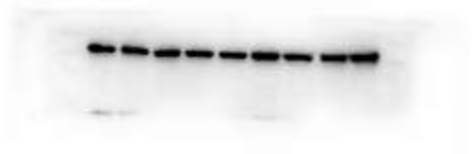

β-actin

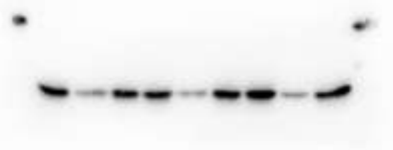

GPX4

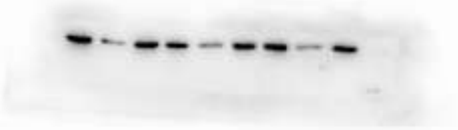

xCT

Supplemental Figure 13G

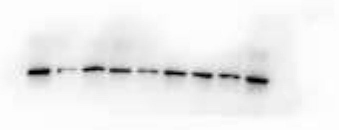

NRF2

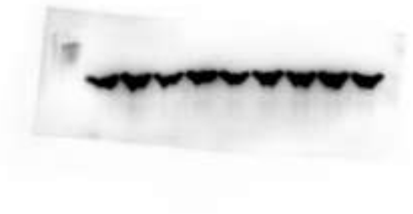

Histone H3

Supplemental Figure 15C

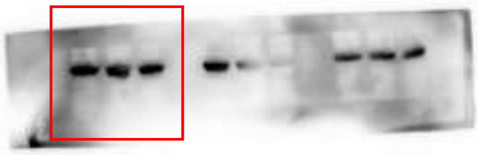

β-actin

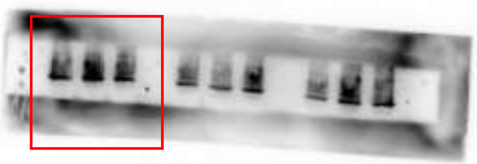

P65

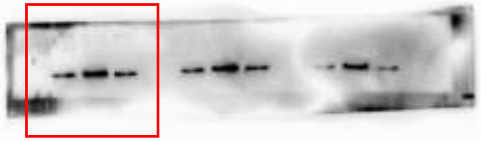

p-P65
